# Development and *In Silico*/*In Vivo* Evaluation of a *Pogostemon cablin* Essential Oil Cream as a Repellent against *Aedes aegypti*

**DOI:** 10.1021/acsomega.6c00802

**Published:** 2026-03-30

**Authors:** Lizandra Lima Santos, Lethicia Barreto Brandão, Alex Bruno Lobato Rodrigues, Rosany Lopes Martins, Anderson Luiz Pena da Costa, Cleidjane Gomes Faustino, Fernando Antônio de Medeiros, Sheylla Susan Moreira da Silva de Almeida

**Affiliations:** † Department of Exact and Technological Sciences, 74364 Federal University of Amapá, 68902280 Macapá, Amapá, Brazil; ‡ Department of Biological and Health Sciences, Federal University of Amapá, 68902280 Macapá, Amapá, Brazil

## Abstract

Mosquito-borne diseases remain a major global public health concern, and the development of safer botanical repellents represents an important complementary strategy to synthetic products. In this study, the essential oil of *Pogostemon cablin* (PCEO) was chemically characterized and incorporated into a topical cream formulation to evaluate its repellent potential against *Aedes aegypti*. Phytochemical analysis by GC-MS revealed patchouli alcohol as the major constituent, consistent with the classical patchouli chemotype. The cream formulation was assessed for physicochemical properties and subjected to *in vivo* repellency testing using an arm-in-cage bioassay, with protection expressed as complete protection time. Molecular docking assays confirmed binding of PCEO constituents to the AaegOBP1 and AgamOBP1 binding sites with α-guaiene and β-elemene showing binding affinities comparable to DEET, supporting their potential contribution to olfactory disruption in mosquitoes. The PCEO cream demonstrated significant repellency, with complete protection time of 180 min. These findings suggest that PCEO-based formulations may represent a promising natural alternative for topical mosquito repellents, integrating phytochemical characterization, formulation design, and mechanistic insights.

## Graphical abstract

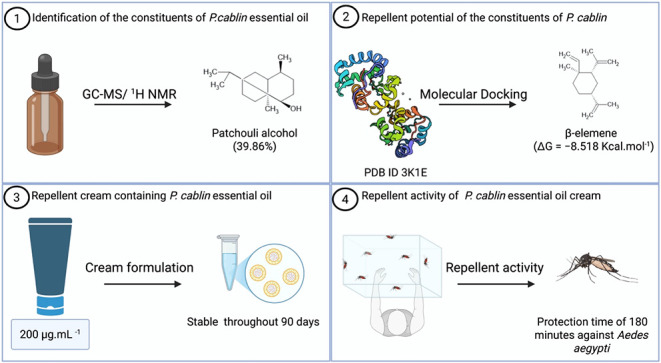

## 1 Introduction

Arboviral diseases transmitted by *Aedes aegypti* (*A. aegypti*), including dengue, Zika, Chikungunya, and yellow fever, remain major global public health challenges due to their increasing incidence and expanding geographic distribution. Climate change, rapid urbanization, and limitations in vector control strategies have intensified transmission dynamics worldwide. In this context, personal protection measures, particularly topical repellents, represent an essential tool to reduce human-vector contact and disease spread.^1^

Although synthetic repellents such as *N*,*N*-diethyl-*m*-toluamide (DEET) are widely used and effective, concerns regarding toxicological safety, environmental persistence, and the potential development of behavioral resistance have stimulated the search for alternative biobased solutions.^2^ Plant-derived essential oils have gained increasing attention due to their chemical diversity, biodegradability, and documented insecticidal and repellent activities.^3^ Additionally, recent reviews have highlighted booth synthetic and plant-based topical repellents as key personal protection tools, emphasizing the need for safer and longer-lasting formulations.^4^

*Pogostemon cablin* (Blanco) Benth. (*P. cablin*), belonging to the Lamiaceae family, is an aromatic plant extensively studied for its rich phytochemical composition. Recent phytochemical investigations report more than 150 identified constituents in its essential oil, predominantly sesquiterpenes and oxygenated terpenes.^5,6^ Patchouli alcohol is consistently described as the major bioactive compound and chemical marker of the species, followed by α-bulnesene, α-guaiene, β-elemene, pogostone, and spathulenol.^5–7^ These metabolites are associated with multiple biological activities, including antimicrobial, anti-inflammatory, antioxidant, and insect-repellent effects.^6,7^

Recent experimental studies have demonstrated that patchouli alcohol and related sesquisterpenes exhibit significant repellent activity against *A. aegypti*, potentially interfering with mosquito olfactory receptors and host-seeking behavior.^8,9^ Despite these advances, investigations correlating detailed phytochemical characterization with repellent efficacy in stable topical formulations remain limited.

Despite the growing evidence of the biological activities of PCEO and its major constituent, patchouli alcohol, studies integrating detailed phytochemical characterization, *in silico* safety prediction, molecular interaction analysis, formulation development and *in vivo* repellency evaluation remain scarce. The lack of integrated approaches combining chemical profiling, computational toxicology and biological validation limits a comprehensive understanding of its potential as a safe and effective topical repellent. Therefore, the present study aimed to evaluate the repellent oil, integrating phytochemical characterization, ADMET prediction, molecular docking and *in vivo* efficacy assays against *A. aegypti*.

## 2 Materials and Methods

### 2.1 Plant Material

*P. cablin* was collected in the district of Fazendinha (00°02′23″ S, 51°06′29″ W), municipality of Macapá, state of Amapá, Brazil. The species was taxonomically identified, and a voucher specimen was deposited in the Amapá Herbarium (HAMAB) of the Scientific and Technological Research Institute of the State of Amapá (IEPA) under registry number 019183.

### 2.2 Essential Oil Extraction

Leaves were dried in a forced-air oven at 36 °C and, after dehydration, ground in an electric mill. The essential oil was obtained by hydrodistillation using a Clevenger-type apparatus at 100 °C for 60 min.^5^ The oil was stored in amber vials under refrigeration at −3 °C until use.

### 2.3 Phytochemical Analysis

#### 2.3.1 Gas Chromatography–Mass Spectrometry (GC–MS)

The chemical composition of the essential oil was determined by GC–MS using a Shimadzu GCMS-QP5050A instrument. Analyses were performed on a DB-5HT capillary column (30 m length, 0.32 mm internal diameter, 0.10 μm film thickness), with nitrogen as the carrier gas, following the conditions described by.^6^ The components were identified by comparing their retention indices (RI) and mass spectra with literature data.^7^ The RI was calculated relative to a number of *n*-alkanes (C8–C40, Sigma-Aldrich, St. Louis, MO, USA) using the Van Den Dool and Kratz equation Retention indices were calculated according to the Van den Dool and Kratz equation using a homologous series of *n*-alkanes (C8–C40, Sigma-Aldrich).^8^

#### 2.3.2 Identification of Major Constituents by ^13^C Nuclear Magnetic Resonance Spectroscopy

The major constituents of the essential oil were confirmed by ^13^C nuclear magnetic resonance (^13^C NMR) spectroscopy. Spectra were on a Bruker AVANCE III HD spectrometer operated at 11.75T, observing ^1^H at 500.13 MHz and ^13^C at 125.77 MHz, equipped with a 5 mm multinuclear BBFO Plus SmartProbe with a *Z*-axis gradient. The ^13^C NMR analysis was performed directly on the crude essential oil without prior fractionation or purification. Samples were dissolved in deuterated chloroform (CDCl_3_) containing tetramethylsilane (TMS) as the internal reference. ^13^C chemical shifts (δ, ppm) were recorded in the 0–220 ppm range.^9^

### 2.4 *In Silico* Predictions for *P. cablin* Molecules

#### 2.4.1 Molecular Structure Building and Geometry Optimization

The molecules identified in the essential oil were drawn using ChemDraw Ultra 12.0 and saved in MDL Molfile (.mol) format. Three-dimensional geometry optimization was performed in ChemSketch using a molecular mechanics (MM) approach with the CHARMM parametrized force field.^10,11^

#### 2.4.2 Prediction of Pharmacokinetic and Toxicological Properties

ADMET properties (absorption, distribution, metabolism, excretion and toxicity) were evaluated using Discovery Studio v16 Discovery Studio v16, San Diego, CA, USA (2013) software.^12–14^ Parameters such as polar surface area (PSA), molecular weight (MW), skin permeability and plasma protein binding were considered to predict therapeutic effectiveness and safety. Toxicity end points included carcinogenicity, mutagenicity (Ames test) and carcinogenic potency (TD_50_), using the TOPKAT module.

#### 2.4.3 Molecular Docking Simulations

Ligand–receptor interactions were investigated using the DockThor platform, configured to evaluate binding based on the MMFF94 force field. Validation of the docking poses considered parameters such as the coordinates of the ligand center, grid box size and overall docking quality.^15,16^ Default algorithm parameters were defined as follows: (1) 24 docking runs per ligand, (2) 1,000,000 evaluations per run and (3) an initial population of 750 individuals. The best protein–ligand complexes were selected based on the root-mean-square deviation (RMSD), with RMSD ≤ 2.0 Å considered acceptable.

### 2.5 Preparation and Preliminary Stability Evaluation of the Cream Formulation

#### 2.5.1 Preparation of the Cream Formulation

A nonionic oil-in-water (O/W) cream was prepared using the phase inversion emulsification technique. The oil phase consisted of cetearyl alcohol, cetearyl alcohol ethoxylate, liquid petrolatum, glyceryl monostearate and BHT. The aqueous phase contained methylparaben, disodium EDTA, propylene glycol and distilled water. The oil and aqueous phases were heated separately to 75 and 85 °C, respectively. The oil phase was then slowly added to the aqueous phase under continuous stirring until the emulsion cooled to 40 °C. The PCEO was incorporated at a concentration of 200 ppm when the system reached approximately 40 °C, after complete homogenization of the phases, to minimize losses due to volatilization. The final formulation was divided into 5 mg aliquots and transferred to Falcon tubes for preliminary stability studies. As a control, samples containing only the base cream (without essential oil) were also prepared and fractionated under the same conditions.^17^

#### 2.5.2 Preliminary Stability Analysis

Formulations were evaluated according to the accelerated stability protocol for cosmetic products,^18^ using centrifugation, thermal stress, light exposure, and assessment of appearance, odor and pH. Centrifugation was performed at increasing speeds (980 to 3000 rpm). Thermal stress tests exposed samples to extreme temperatures (5 and 45 °C). Photostability was assessed by monitoring changes in color after direct light exposure. Macroscopic appearance was evaluated by the absence of phase separation, precipitation or turbidity. Odor was compared with that of the control cream (without essential oil). For pH determination, a 10% (w/w) aqueous dispersion of the cream was prepared, and values between 5.5 and 6.5 were considered compatible with normal skin pH. These criteria were used to confirm the preliminary stability of the formulations

### 2.6 Evaluation of Repellent Activity

Repellent activity was evaluated following the standard protocol recommended by the World Health Organization.^19^ For the bioassays, 50 adults female *A. aegypti* (5–7 days old) were maintained in cages (40 × 40 × 40 cm^3^), without blood feeding and deprived of 10% sucrose solution for 24 h prior to the experiments.

Healthy volunteers of both sexes, aged 20–35 years, with no history of allergic reactions to mosquito bites or dermatological diseases, were recruited. Before testing, volunteers were instructed not to use moisturizers or other topical products for 12 h. During the assays, the hands and arms of the participants were protected with plastic gloves, while the forearms were exposed inside cages containing *A. aegypti* under controlled conditions of temperature (25 ± 2 °C) and relative humidity (80 ± 5%). An area of 600 cm^2^ on the forearm was treated with 1 mL of the test sample (200 μg/mL). The positive control consisted of a commercial DEET-based repellent applied to the right forearm according to the manufacturer’s instructions. The negative control was 1% Tween 80.

Experiments were carried out on different days, with six 30 min replications. During each interval, the number of mosquitoes landing or biting in each experimental condition was recorded, allowing calculation of the protection efficacy of the samples.

### 2.7 Statistical Analysis

Bioassay results were expressed as means ± standard deviation and summarized in tables and graphs, following statistical reporting approaches commonly adopted in experimental bioassays and analytical studies.^20,21^ Differences between treatments were assessed by one-way ANOVA using SPSS (Statistical Package for the Social Sciences), adopting a significance level of 5% (*p* ≤ 0.05).

### 2.8 Ethical Aspects

This study was approved by the Research Ethics Committee (CEP) of the Federal University of Amapá, in accordance with Resolution 466/12 of the Brazilian National Health Council (CNS) as per the Certificate of Presentation for Ethical review (CAAE) number 43618621.3.0000.0003 and opinion 4.621.893 available on the Brazil Platform.^22^ All ethical principles were strictly followed, including nondiscrimination in participant selection, avoidance of unnecessary risks, and the guarantee of legitimacy, privacy and confidentiality of information. The research procedures were initiated only after all participants had signed the informed consent form. The results will be disseminated in a manner that fully respects these ethical principles.

## 3 Results and Discussion

### 3.1 Phytochemical Analysis

#### 3.1.1 Gas chromatography–Mass Spectrometry and Nuclear Magnetic Resonance

Phytochemical analysis of PCEO by gas chromatography coupled to mass spectrometry (GC–MS) led to the identification of 16 compounds, mainly sesquiterpenes and oxygenated terpenes. Among the identified constituents, patchouli alcohol (Figure 1) was the major component, with a relative abundance of 39.83% (Table 1).

**Figure 1:**
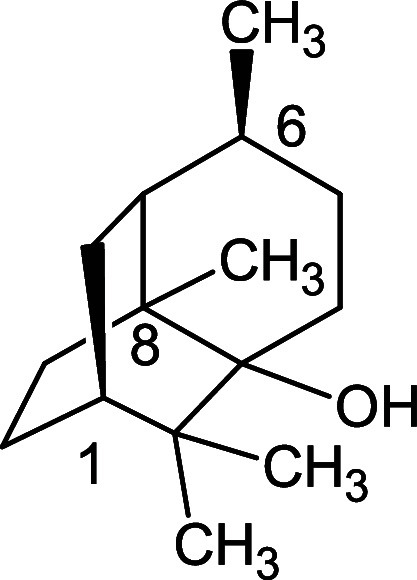
Molecular structures of the major constituents of PCEO.

**Table 1:** Compounds Identified in the GC–MS Analysis of PCEO^a^

No.	*R*_t_ (min)	RI*	compounds	relative percentage
1	26.121	1432	β-patchoulene	3.43%
2	26.631	1398	β-elemene	1.12%
3	27.235	1474	cycloseychellene	0.60%
4	27.790	1494	(*E*)-caryophyllene	3.37%
5	28.779	1490	**α-guaiene**	**11.35%**
6	29.340	1403	*(E)*-γ-bisabolene	5.17%
7	30.118	1490	**α-bulnesene**	**14.01%**
8	30.643	1461	γ-patchoulene	0.83%
9	31.074	1490	aciphyllene	3.04%
10	33.245	1533	norpatchoulenol	2.46%
11	34.247	1507	caryophyllene oxide	0.66%
12	36.509	1530	Palustrol	1.13%
13	36.720	1536	Spathulenol	0.60%
14	37.785	1420	**patchouli alcohol**	**39.83%**
15	38.174	1431	Allohimachalol	0.71%
16	39.707	1453	**Pogostone**	**9.98%**
Compounds identified				98.29%

a IR = Van den Dool and Kratz^8^ retention index; tR = retention time.

Among the identified compounds, patchouli alcohol was present at the highest abundance and was therefore considered the major constituent. The ^13^C NMR spectrum of the crude essential oil exhibited characteristic sgnals consistent with oxygenated sesquiterpenes, particularly patchouli alcohol. A diagnostic resonance around δ 75 ppm, corresponding to the oxygenated carbon (C–OH), supports the presence of patchouli alcohol as the major constituent. Additional signals in the olefinic region (δ 110–150 ppm) were consistent with guaiene-and-bulnesene-type sesquiterpenes, corroborating the GC-MS results. Overall, the spectral profile was in agreement with literature data for the classical patchouli chemotype.^23,24^ The complete ^13^C NMR spectral data and comparative values are provided in the Supporting Information.

Several characteristic constituents of PCEO were detected, including patchouli alcohol, β-patchoulene, patchoulene epoxide, pogostone and pachypodol, in agreement with previous reports.^25,26^ Santos et al. identified 29 volatile compounds in the essential oil of this species, highlighting patchouli alcohol (33.25%), seychellene (6.12%), α-bulnesene (4.11%), pogostol (6.33%) and norpatchoulenol (5.72%) as major constituents.^27^

Similarly, Liu et al.^28^ reported 23 constituents in PCEO, with patchouli alcohol (41.31%), pogostone (18.06%), α-bulnesene (6.56%), caryophyllene (5.96%) and seychellene (4.32%) as the predominant components. Pandey et al. also found patchouli alcohol (44.52%) and caryophyllene (12.86%) as major constituents in PCEO collected in India.^29^ Despite quantitative variations, these studies consistently indicate patchouli alcohol as the predominant compound, corroborating the present results.

More than 150 constituents have been reported in PCEO,^25^ and its composition can vary considerably as a function of environmental factors, such as edaphic conditions and genotype, which influence both the diversity and levels of its constituents.^30,31^ Nevertheless, patchouli alcohol and α-patchoulene, together with their stereoisomers, remain the compounds most frequently associated with the pharmacological properties of this species.^32,33^

Studies conducted in different regions further confirm that cultivation conditions affect the chemical profile of PCEO. Zhang demonstrated statistically significant differences in the composition of plants grown in distinct locations in China, which were attributed to local edaphic conditions.^30^ However, even in the presence of such variation, patchouli alcohol consistently remained the major constituent, in agreement with the composition identified in the present work.

Although GC–MS is considered the method of choice for profiling the volatile fraction of essential oils, identification based solely on retention indices and mass fragmentation patterns can be challenging in the presence of structural isomers, such as α- and β-patchoulene, α- and β-guaiene, or different guaiane- and bulnane-type sesquiterpenes, which often display very similar mass spectra.^2,25^

In this context, ^13^C NMR spectroscopy provides a complementary approach, delivering unambiguous information about the chemical environment of each carbon and allowing confident confirmation of the structures proposed from GC–MS data. Comparison of the experimental chemical shifts obtained for α-guaiene, α-bulnesene, patchouli alcohol and pogostone with literature values showed excellent agreement, with differences below 0.3 ppm, confirming that the essential oil follows the classical *P. cablin* chemotype, characterized by high levels of oxygenated sesquiterpenes.^34–36^

Thus, structural confirmation of the main sesquiterpenes by GC–MS and NMR ensures that the molecules submitted to *in silico* analyses accurately represent the true chemical composition of the essential oil used. This, in turn, allows ADMET predictions and docking simulations to be interpreted consistently in light of the experimentally characterized chemical profile.

### 3.2 *In Silico* Study of *P. cablin* Molecules

#### 3.2.1 Pharmacokinetic and Toxicological Predictions

The 16 molecules identified in PCEO by GC–MS and NMR, together with the reference compounds DEET and esbiothrin, were subjected to pharmacokinetic predictions using the DS-ADMET model. Although the formulation developed is intended for topical use, ADMET prediction is essential as a first step in safety assessment. Parameters such as intestinal absorption (IA) and blood–brain barrier (BBB) penetration were used to estimate the potential for systemic exposure if small amounts of the compounds cross the skin barrier. Molecules with high predicted permeability or potential BBB penetration may pose a risk of systemic effects, including neurotoxicity, even when applied topically.^14^

In this context, the results showed that all analyzed molecules fell within the 95% and 99% confidence ellipses for the PSA and ALogP98 descriptors, as illustrated in Figure 2, indicating a low risk of crossing critical biological barriers. These descriptors estimate the likelihood of systemic distribution following topical application and therefore help to anticipate potential alerts related to neurotoxicity or bioaccumulation.^37,38^

**Figure 2:**
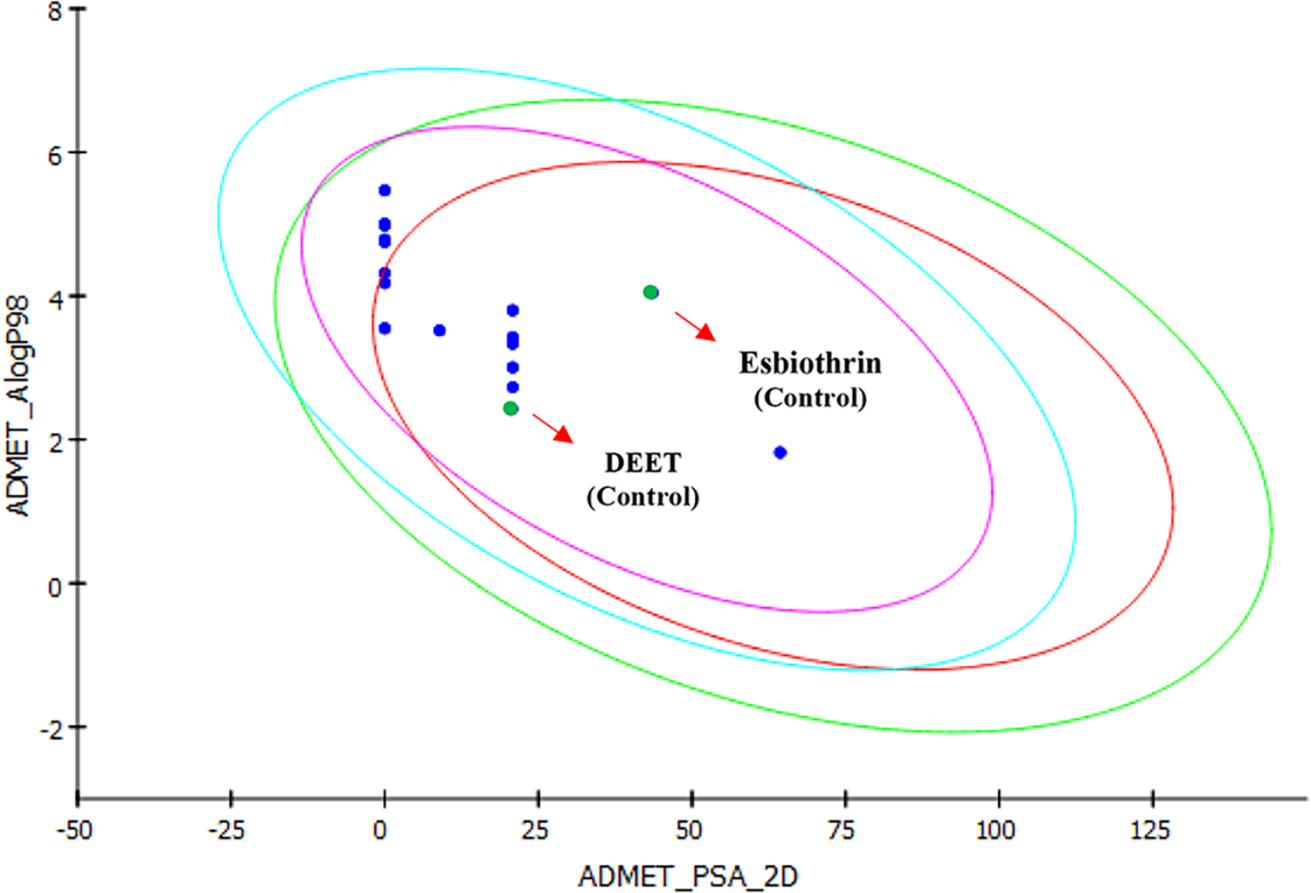
Polar surface area (PSA) *versus* *A* log *P* plot showing the molecules within the 95% and 99% confidence limit ellipses for the blood–brain barrier (BBB) and intestinal absorption (IA).

Considering standard absorption, distribution, metabolism and excretion (ADME) reference parameters, the essential-oil constituents met the main pharmacokinetic criteria assessed, including permeability (PSA and ALogP), aqueous solubility, plasma protein binding (PPB), potential CYP2D6 inhibition, predicted systemic absorption and BBB penetration. These indicators follow the classical rules of Lipinski, Ghose and Veber, widely used for evaluating physicochemical properties and predictive safety.^39^ Few or no violations of these rules suggest that the molecules exhibit structurally favorable characteristics from a pharmacokinetic standpoint, supporting the inference of a safe behavior in scenarios of potential systemic exposure. The computational parameters obtained for the molecules and controls are summarized in Table 2.

**Table 2:** Pharmacokinetic (ADME) Computational Parameters for the Molecules Identified in PCEO^a^

molecules	PPB	hepatotoxicity	CYP2D6 binding	solubility	BBB	IA
Deet	True	False	False	3	1	0
Esbiothrin	True	False	True	2	1	0
Allohimachalol	True	False	False	2	1	0
α-bulnesene	True	False	False	2	0	1
β-Elemene	True	False	False	2	0	1
β-patchoulene	True	True	False	2	0	0
Caryophyllene Oxide	True	True	False	2	0	0
(*E*)-caryophyllene	True	False	False	2	0	1
(*E*)-γ–bisabolene	True	False	True	2	0	1
Cycloseychellene	True	True	False	2	0	0
γ-patchoulene	True	False	False	2	0	0
Norpatchoulenol	True	False	False	3	1	0
Palustrol	True	True	False	2	1	0
Pogostona	False	True	False	3	3	0
Spathulenol	True	True	False	3	1	0
Aciphyllene	True	False	False	2	0	1
Patchouli Alcohol	True	True	False	2	1	0
α-guaiene	True	False	False	2	0	1

a BBB—blood–brain barrier (0 = very high penetration; 1 = high; 2 = medium; 3 = low; 4 = very low). IA—human intestinal absorption (acceptable range: 0–2, where 0 indicates good absorption). Aqueous solubility (acceptable range: 0–3, where 3 indicates good solubility). CYP2D6 binding—cytochrome P450 (CYP450) 2D6 inhibition (false = noninhibitor; true = inhibitor). PPB—plasma protein binding (false = does not bind to plasma proteins; true = binds to plasma proteins).

Overall, the molecules displayed aqueous solubility and intestinal absorption values within standard ranges when compared with the reference compounds. However, esbiothrin and (*E*)-γ-bisabolene showed an alert for CYP2D6 binding, indicating possible interactions with cytochrome P450 that may involve enzyme conformational changes or additional mechanisms beyond electron transfer.^40^

β-Patchoulene, caryophyllene oxide, cycloseychellene, palustrol, pogostone, spathulenol and patchouli alcohol showed hepatotoxicity alerts, possibly due to the presence of toxicophoric groups that could cause liver damage under prolonged exposure.^38^ Among them, pogostone was the only molecule that did not bind to plasma proteins, a factor that can influence metabolic reactions and stereochemical changes.^41^ These effects may be exacerbated in the presence of enzymes or abrupt pH variations, emphasizing the need for additional studies to evaluate the safety of this compound under long-term use.

The hepatotoxicity and CYP2D6-binding alerts for the aforementioned molecules should be interpreted with caution, as they refer to systemic metabolism, which would only be relevant under conditions of significant dermal absorption—a scenario considered unlikely due to their relatively high molecular weight and low deep dermal penetration.^42,43^ Nonetheless, these findings are valuable as an initial safety-screening step.

The TOPKAT software, developed by the U.S. Food and Drug Administration (USFDA), was used to predict the toxicological risk of the molecules. This tool identifies specific structural fragments within a molecule that may be associated with potential toxic effects.^44^ Detailed results of the toxicological predictions, including carcinogenicity, mutagenicity and skin irritation, are presented in Table 3.

**Table 3:** Computational Parameters for USFDA Rodent Carcinogenicity, Ames Mutagenicity, Skin Sensitization and Skin Irritancy

molecules	mouse male	mouse female	ames mutagenicity	skin sensitization	skin irritancy
Deet	Non-Carcinogen	Multi-Carcinogen	Non-Mutagen	None	None
esbiothrin	Multi-Carcinogen	Multi-Carcinogen	Non-Mutagen	Weak	Mild
allohimachalol	Multi-Carcinogen	Multi-Carcinogen	Non-Mutagen	Weak	None
α-bulnesene	Multi-Carcinogen	Multi-Carcinogen	Non-Mutagen	Weak	None
β-elemene	Multi-Carcinogen	Multi-Carcinogen	Non-Mutagen	Weak	None
β-patchoulene	Multi-Carcinogen	Multi-Carcinogen	Non-Mutagen	Weak	None
caryophyllene oxide	Multi-Carcinogen	Multi-Carcinogen	Non-Mutagen	Weak	Mild
(*E*)-caryophyllene	Multi-Carcinogen	Multi-Carcinogen	Non-Mutagen	Weak	None
(*E*)-γ −bisabolene	Single-Carcinogen	Multi-Carcinogen	Non-Mutagen	Weak	None
cycloseychellene	Multi-Carcinogen	Multi-Carcinogen	Non-Mutagen	Weak	Severe
γ-patchoulene	Multi-Carcinogen	Multi-Carcinogen	Non-Mutagen	Weak	None
norpatchoulenol	Multi-Carcinogen	Multi-Carcinogen	Non-Mutagen	Weak	Severe
palustrol	Multi-Carcinogen	Multi-Carcinogen	Non-Mutagen	Weak	Severe
pogostona	Multi-Carcinogen	Multi-Carcinogen	Non-Mutagen	Weak	Mild
Spathulenol	Multi-Carcinogen	Multi-Carcinogen	Non-Mutagen	Weak	Moderate
aciphyllene	Single-Carcinogen	Multi-Carcinogen	Non-Mutagen	Weak	None
patchouli alcohol	Multi-Carcinogen	Multi-Carcinogen	Non-Mutagen	Weak	Severe
α-guaiene	Multi-Carcinogen	Multi-Carcinogen	Non-Mutagen	Weak	None

According to Table 3, all molecules evaluated in TOPKAT displayed some level of carcinogenicity alert, classified as single- or multicarcinogen, including DEET, esbiothrin and the 16 molecules identified in PCEO. However, proper risk assessment depends on additional studies using specific dose metrics.^45–50^ In contrast, Ames mutagenicity predictions were negative for all compounds, supporting the use of this assay as a globally recognized first-line tool for screening the mutagenic potential of new chemical entities.^51–54^

Regarding skin sensitization, none of the molecules showed a significant risk. For skin irritation, however, cycloseychellene, norpatchoulenol, palustrol and patchouli alcohol presented high-risk (severe) alerts. With respect to carcinogenic potency, only seven molecules exhibited TD_50_ values higher than the reference control (TD_50_ = 100,95 mg/kg/day), indicating an overall low carcinogenic potential for the set of molecules analyzed. Detailed data are given in Table 4, reinforcing the potential of PCEO constituents for safe use at appropriate doses.

**Table 4:** Carcinogenic Potency (TD_50_) for the Selected Molecules

	carcinogenic potency TD_50_ (mg/kg body weight/day)		
molecules	mouse	rat	rat maximum tolerance dose
Deet	183.726	82.9772	6516.93
esbiothrin	120.659	16.6586	100.95
allohimachalol	37.0228	11.0333	155.103
α-bulnesene	29.2399	13.8252	152.533
β-Elemene	18.3669	100.371	220.829
β-patchoulene	5.18701	8.59857	506.407
caryophyllene oxide	4.59107	5.36247	30.6402
(*E*)-caryophyllene	23.5577	36.5708	71.0585
(*E*)-γ −bisabolene	61.2116	99.9317	493.082
cycloseychellene	1.78749	1.88338	789.664
γ-patchoulene	5.33837	6.82919	166.15
norpatchoulenol	9.0603	11.0298	273.522
palustrol	4.6333	3.80095	340.00
pogostona	463.283	120.139	156.127
spathulenol	8.79074	7.51001	123.72
aciphyllene	29.2399	13.8252	152.533
patchouli alcohol	4.53998	3.73545	322.817
α-guaiene	19.4027	13.8252	152.533

The carcinogenic potency data indicated that allohimachalol, α-bulnesene, β-elemene, γ-patchoulene, norpatchoulenol, aciphyllene and α-guaiene exhibited TD_50_ values higher than those of the control compounds, highlighting their lower carcinogenic potential. Conversely, molecules such as β-patchoulene, (*E*)-γ-bisabolene, cycloseychellene, palustrol, pogostone, spathulenol and patchouli alcohol, although also displaying relatively high TD_50_ values, were associated with hepatotoxicity alerts, underscoring the need for careful consideration under long-term exposure. TD_50_, an analogue of LD_50_, represents the daily dose required to induce tumors in 50% of test animals. Low TD_50_ values indicate more potent carcinogens, whereas higher values suggest lower carcinogenic potential. TD_50_ can be calculated for specific tumor types, tissues or combinations thereof.^47,48,54^

Taken together, the pharmacokinetic and toxicological predictions were in direct agreement with the repellent activity observed for the topical formulation containing PCEO (Section 3.3). The molecules identified by GC–MS and confirmed by NMR—particularly patchouli alcohol, α-guaiene, α-bulnesene and β-elemene—showed favorable predicted permeability, low potential for systemic toxicity and lack of CYP2D6 inhibition in the ADMET models. These parameters are highly relevant in the context of topical use, indicating a safety profile compatible with dermal formulations and suggesting a low risk of adverse effects in the event of transcutaneous absorption.

#### 3.2.2 Molecular Docking

The molecular docking protocol was first validated to ensure its reliability. The procedure involved the structural superposition of the binding sites of the odorant-binding protein 1 of *A. aegypti* (AaegOBP1, PDB ID 3K1E) and the odorant-binding protein 1 of *Anopheles gambiae* (AgamOBP1, PDB ID 3N7H), as well as the crystallographic ligand of AgamOBP1. Validation results were considered satisfactory, since the relative positions of the crystallographic ligand and the docked ligand showed high similarity (Figure 3).

**Figure 3:**
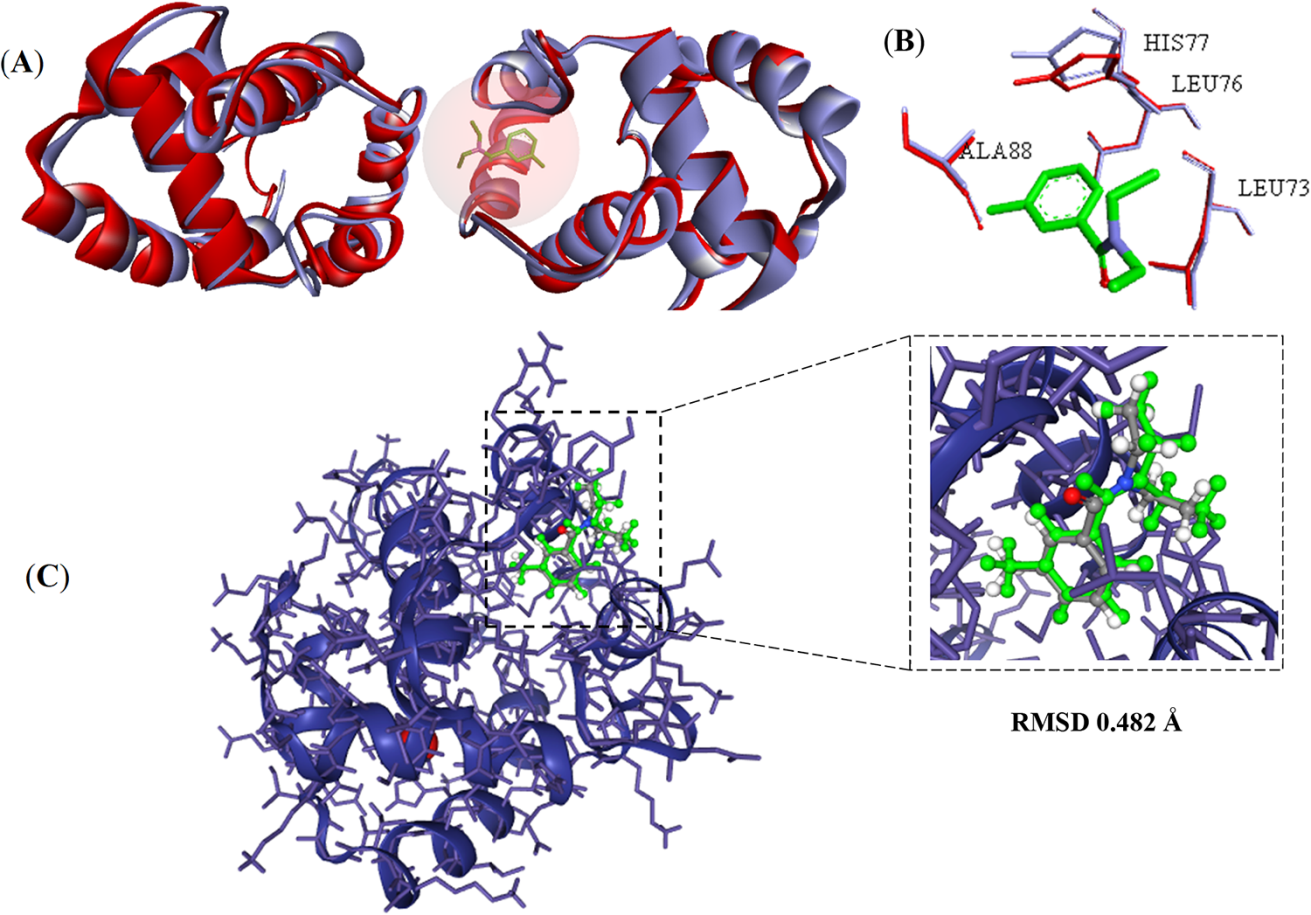
Global and local structural alignment of AaegOBP1 (lilac) and AgamOBP1 (red) (A) and superposition of binding-site residues (B). Validation of the molecular docking protocols (C) for the crystallographic structure of AgamOBP1.

After protonation, optimization and energy minimization of AaegOBP1, structural superposition and alignment were performed. The root-mean-square deviation (RMSD) for amino acid residues in the AaegOBP1 binding site was 0.267 Å, while the RMSD for the atoms of the crystallographic ligand and the docked ligand in the AgamOBP1 model was 0.482 Å.

Based on the cross-docking study reported by de Araújo Neto et al., the structures of AgamOBP1 (PDB ID 3N7H) and AaegOBP1 (PDB ID 3K1E) were compared.^55^ The analysis revealed 82% of global sequence identity and 100% identity in the binding-site residues. This high similarity enabled the definition of the active site of AaegOBP1 and validation of the molecular docking protocol using DEET, the crystallographic ligand of AgamOBP1, as a reference. In addition, atomic deviations were low (RMSD = 0.267 Å), confirming the accuracy of the model.

It is important emphasize that odorant-binding proteins are essential components of the mosquito olfactory system and facilitate the transport of semiochemicals to olfactory receptors. Interference with OBP-ligand interactions has been proposed as a molecular mechanism underlying repellent action.^56–58^ In this context, the favorable binding interactions observed between patchouli alcohol and AaegOBP1 may suggest a potential capacity to disrupt odorant recognition pathways, complementing the *in vivo* repellency findings.

In this context, for AaegOBP1 complexed with DEET, interactions similar to those observed for β-elemene were identified. β-Elemene engaged residues located around the α-helix, including Leu72, Leu88, His76, Ala88 and Leu95, as illustrated in Figure 4. These interactions support the potential of β-elemene as a functional ligand at the same binding site as DEET.

**Figure 4:**
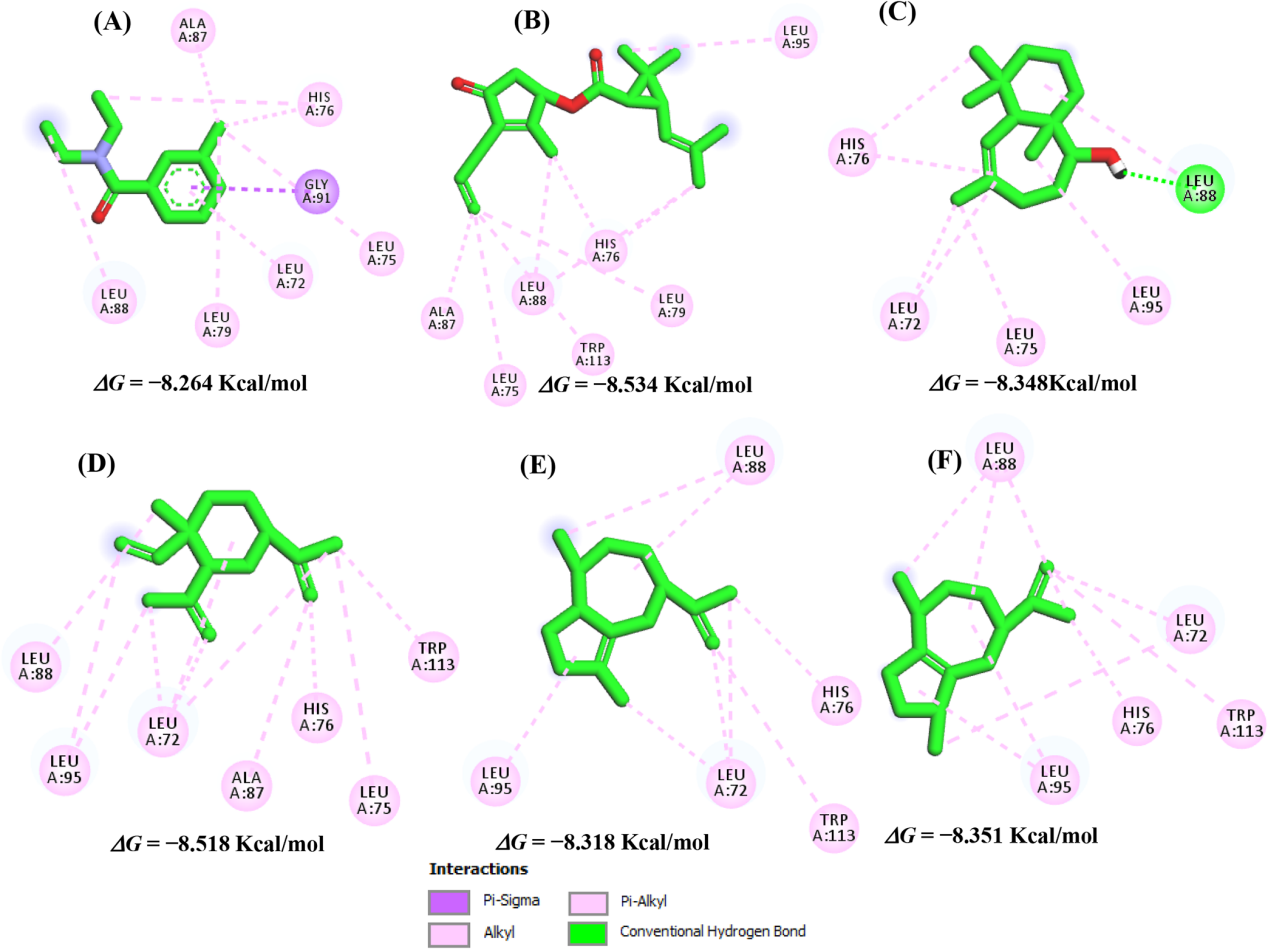
Interactions of the controls DEET (A) and esbiothrin (B), and the potential repellent agents allohimachalol (C), β-elemene (D), aciphyllene (E) and α-guaiene (F) in the AaegOBP1 binding site.

*In silico* studies have shown that DEET interacts predominantly through hydrophobic contacts with residues Leu96, Leu73, His77, Asp82, Leu89 and Lys93.^58^ Comparing these interactions with the crystallographic ligand maps, DEET also formed additional hydrophobic contacts with Phe59, Leu76, Phe123, Trp114 and Tyr122.^57^ These results are consistent with the literature, as the residues involved in DEET binding in our docking model (Figure 3A) correspond—after alignment of AaegOBP1 and AgamOBP1—to the equivalent residues Leu72/Leu73, Leu75/Leu76, Leu88/Leu89 and His76/His77 (Figure 2A).

β-Elemene exhibited a binding affinity differing by only ± 0,254 kcal/mol from DEET, which supports its classification as a potential repellent agent due to the high similarity of its interactions with the amino acid residues in the binding site. Likewise, α-guaiene engaged the AgamOBP1 receptor in a manner similar to DEET, establishing interactions with residues Leu73, Leu76, His77 and Ala89 around the α-helix,^56^ as illustrated in Figure 5.

**Figure 5:**
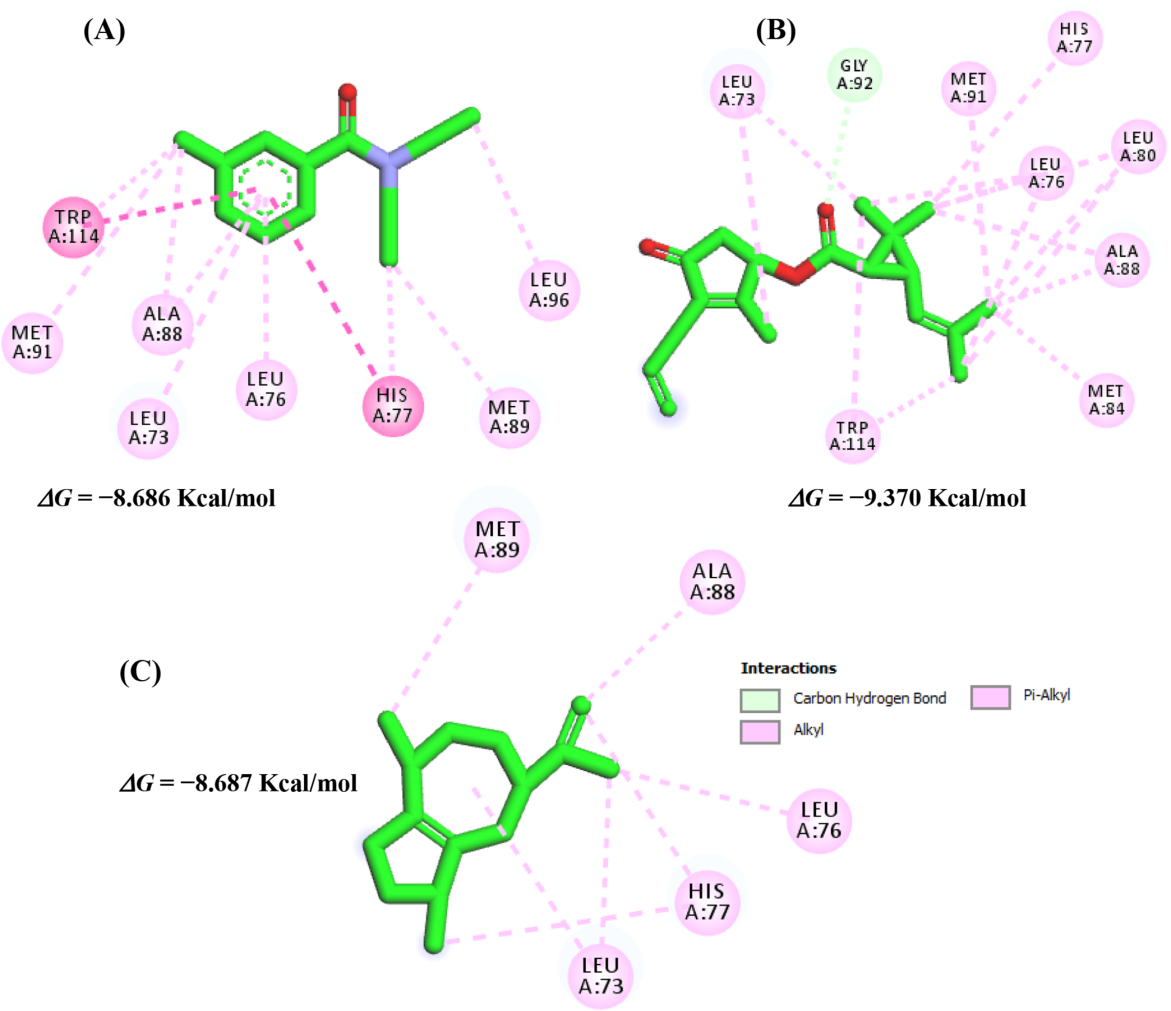
Interactions of the controls DEET (A) and esbiothrin (B), and the potential repellent agent 9 (C) with the binding site of AgamOBP1.

The difference in binding affinity between α-guaiene and DEET was only ±0.001 kcal/mol, further reinforcing its potential as a repellent due to the strong similarity of interactions in the binding pocket. These findings highlight the feasibility of β-elemene and α-guaiene as functional alternatives to DEET and suggest that these sesquiterpenes may contribute to olfactory disorientation in mosquitoes, which is consistent with the high protection rates observed in the repellency assays.

Therefore, the *in silico* predictions not only support the mechanistic plausibility of the repellent effect but also help to explain, at the molecular level, why the formulation containing PCEO afforded 100% protection up to 180 min. Overall, the chemical composition, molecular modeling and biological assays form a coherent body of evidence supporting the potential of PCEO as an effective repellent active ingredient with an acceptable preliminary safety profile.

### 3.3 Development and Preliminary Stability Assessment of the Cream Formulation

The cream formulation exhibited stable organoleptic characteristics throughout 90 days of monitoring. No changes in color, odor, appearance or phase separation were observed under any of the storage conditions tested (room temperature, refrigeration and oven). The pH values ranged between 4.5 and 5.9, remaining within the range considered compatible with human skin. Centrifugation did not induce instability in any sample, indicating good resistance to movement and physical stress. Exposure to light radiation also did not result in perceptible visual changes, reinforcing the preliminary stability of the cream (Table 5).

**Table 5:** Preliminary Stability Evaluation of the PCEO Cream Formulation^a^

days	room temperature	refrigerated temperature	centrifugation	oven	light radiation	pH
0	N	N	no phase separation	N	N	4.5
3	N	N	no phase separation	N	N	4.8
6	N	N	no phase separation	N	N	5.3
9	N	N	no phase separation	N	N	5.5
12	N	N	no phase separation	N	N	5.5
15	N	N	no phase separation	N	N	5.8
30	N	N	no phase separation	N	N	5.9
60	N	N	no phase separation	N	N	5.9
90	N	N	no phase separation	N	N	5.7

a N = normal.

Throughout the evaluation period, all samples remained physically stable, with no evidence of phase separation, precipitation or turbidity. The thermal stress test indicated that the formulation was stable at low temperatures (2–5 °C) as well as at elevated temperature (45 °C). These results confirm that the cream presents adequate resistance under simulated environmental conditions, without signs of structural degradation or loss of functionality.

An increase in pH was observed over time. Skin pH typically falls between 5 and 6 under normal conditions and may vary according to skin tone/color, sun exposure and certain skin diseases. It is well established that topical products can modify skin pH; therefore, the formulation pH should be compatible with the skin in order to ensure good tolerability.^59^ In the present cream, the pH ranged from 5.0 to 5.9, *i.e.*, within the tolerability range and easily buffered by the skin.

Cosmetic formulations containing PCEO have shown stability profiles similar to that observed here. Isnaini et al. evaluated a body butter containing patchouli oil under accelerated stability conditions over seven cycles, monitoring appearance, homogeneity, pH, spreadability, adhesion and absorption capacity.^60^ No significant changes in physical properties were observed, indicating good resistance to temperature variation and prolonged storage.

In a subsequent study, the same author developed an O/W cream containing PCEO and *Moringa oleifera* oil, also subjected to heating–cooling cycles and evaluation of pH, viscosity and organoleptic characteristics. The formulations maintained pH values within the acceptable range for cutaneous use and did not exhibit phase separation or perceptible color changes.^57^

Similar results were reported by Nurhadianty et al. for a moisturizing lotion designed for tropical skin and containing essential oils, including *P. cablin*. The product remained stable after seven 4 °C/40 °C cycles, maintaining pH and consistency within the specified limits for dermatological lotions.^61^

Additional evidence supporting the technological feasibility of patchouli-based topical systems was provided by Fazariyana and Indri, who optimized a lotion formulation containing PCEO and evaluated both is physicochemical stability and sun protection factor (SPF). The study demonstrated that formulation optimization-particularly regarding emulsifier concentration and oil phase proportion-was crucial to achieving homogeneous systems with acceptable pH, viscosity, and spreadability profiles. Importantly, the optimized lotion exhibited stable characteristics under storage conditions and provided measurable SPF values, highlighting the multifunctional potential of PCEO in dermocosmetic applications.^59^ These findings reinforce that properly designed O/W systems can preserve the stability of volatile terpenoid constituents while maintaining functional performance, in agreement with the preliminary stability behavior observed for the PCEO cream in the present study.

Overall, these studies reinforce that O/W emulsions containing PCEO can achieve adequate stability when the composition of the oil phase and the emulsifying system is properly optimized, in agreement with the behavior observed for the PCEO cream in the present study, and demonstrating the technological feasibility of the proposed formulation.

### 3.4 Repellent Activity of PCEO-Based Cream Formulations

The repellent activity of the cream formulation containing PCEO was assessed based on the mean protection time, using arm-in-cage contact tests at 30 min intervals over 180 min. An initial concentration of 100 ppm of the cream formulation provided low protection, which led to the testing of a 200-ppm concentration. At 200 ppm, the formulation achieved 100% protection for 180 min (Figure 6).

**Figure 6:**
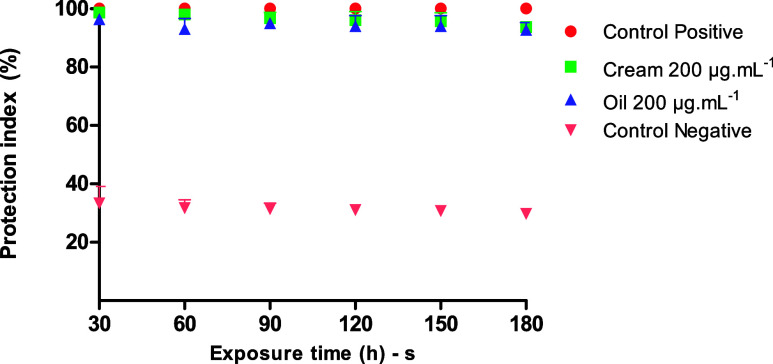
Repellency test of the cream formulation containing PCEO against *A. aegypti* females.

The repellency performance observed in the present study should be interpreted considering standardized evaluation criteria. The arm-in-cage bioassay is widely employed for assessing topical mosquito repellents under controlled exposure conditions, and complete protection time is regarded as a practical and reproducible parameter for comparing formulations and active ingredients.^62^ However, complete protection time values are influenced not only by intrinsic biological activity but also by formulation-dependent factors such as evaporation rate, film formation on the skin surface, and retention of volatile constituents. Therefore, the protection time recorded for PCEO cream reflects the combined effect of chemical composition and release behavior from the topical matrix.

ANOVA showed that both main effects and their interaction were highly significant (treatment: *F*(3,1176) = 56.098, *p* < 0.0001; time: *F*(5,1176) = 183.9, *p* < 0.0001; interaction: *F*(15,1176) = 85.18, *p* < 0.0001). These results indicate that the type of treatment was by far the main determinant of repellency, explaining approximately 98% of the total variability in the data. In practical terms, this means that differences in repellency were predominantly driven by the nature of the treatments (cream with PCEO, DEET and controls), rather than by the duration of exposure within the time window evaluated.

Many natural products exhibit repellent properties against a wide range of pests and insects, and several plant-derived oils have been identified as promising botanical sources of insect repellents.^3,4^ Essential oils from numerous plant species have been tested against different mosquito species worldwide.^1,6,27,28^ However, the repellent activity of essential oils against vector mosquitoes varies substantially due to factors such as the plant part used, intrinsic plant properties, environmental conditions, distillation method, and the species and age of the target mosquitoes^2,5,29,61^

The significant repellency provided by plant-derived products is closely related to their chemical composition. According to Taupik et al.^34^ plants rich in terpenes can modulate insect biochemical and physiological processes. In this context, patchouli alcohol, a major sesquiterpene present in PCEO, has been evaluated as an isolated compound for repellent potential. At a dose of 2 mg/cm^2^, it was the most effective, providing 100% protection against *A. aegypti* for up to 280 min.^63^ These findings indicate that the major component of *P. cablin* exhibits marked repellency at relatively low concentrations and with a considerable protection time.

Recent findings by Yunus et al. further reinforce the repellent potential of PCEO derived from different regional varieties. The outhors demonstrated that PCEO from Southeast Sulawesi exhibited significant protection against *A. aegypti*, with 90.44% protection for the lotion containing 10% PCEO. In this context, the high repellency observed for the present PCEO formulation may be partly attributed to its chemical profile consistency with classical patchouli chemotypes.^3^

Similary, Ariati investigated the formulation of a mosquito repellent spray combining citronella oil (*Cymbopogon nardus*) and PCEO, reporting superior protective efficacy compared to the individual oils. LSD analysis showed that the concentration presented a good level of effectiveness as a mosquito repellent, with a concentration of citronella oil of 10% and PCEO OF 6% showed a mosquito repellent power percentage of 92%. These findings corroborate the concept that botanical repellents can benefit from multicomponent formulations, in which PCEO contributes not only as an active repellent but also as a fixative agent capable of modulating the evaporation rates of more volatile compounds. This formulation-based synergy supports the interpretation that repellency is influenced by both intrinsic chemical activity and vehicle-mediated performance.^64^

Complementarily, Widawati and Riandi^65^ explored the synergy among different constituents by testing the repellency of a topical lotion based on patchouli oil combined with betel oil against *A. aegypti*. Efficacy was determined by the ability of the formulation to prevent yellow fever mosquitoes from biting human forearms and expressed as a protection percentage. The modified patchouli leaf lotion provided more than 90% protection for 360 min, an effect possibly related to the association of the two oils, which may have potentiated the formulation.

Regarding the mechanism of repellency, it is known that insects detect odors through odorant receptors (ORx) that form complexes with the coreceptor Orco. Odor-detecting sensory neurons are activated when an odorant binds to ORx, triggering the opening of Orco ion channels. Consequently, allosteric agonists and antagonists that target ORx and Orco can act as potential repellents.^66,67^ Thus, attributed to the high content of oxygenated sesquiterpenes—or their combined synergistic effects—may interfere with odor detection, thereby promoting repellency.^5,65^

Further evidence supporting the relevance of repellency formulation parameters was provided by Sutthanon et al. (2026), who developed and evaluated a novel topical topical lotion containing 15%w/w of a mixture of citronella, patchouli (PCEO), and sage essential oils, with emphasis on both skin safety and repellent efficacy. The lotion maintained >90% repellency against *A. Aegypti* for up to 3 h, demonstrating that optimized essential-oil blends incorporated into stable lotion systems can provide sustained protection.^68^

These observations are consistent with the present findings, suggesting that formulation design may compensate for lower active load through improved retention and controlled diffusion.Overall, the repellent performance of the cream formulation, together with the *in silico* and phytochemical data, supports the potential use of PCEO as an effective and safe active ingredient in topical mosquito-repellent products.

## 4 Conclusions

Patchouli alcohol was identified as the major constituent of PCEO, followed by α-bulnesene, α-guaiene and pogostone. However, the higher abundance of these compounds does not necessarily imply that the pharmacological or repellent effects are exclusively attributable to them. The *in silico* ADMET study indicated that molecules such as allohimachalol, α-bulnesene, β-elemene, (*E*)-caryophyllene, norpatchoulenol, aciphyllene and α-guaiene display favorable absorption profiles and low predicted toxicity, supporting their potential as safe bioactive candidates. Patchouli alcohol, the major constituent of PCEO, also showed a favorable absorption profile and an acceptable predicted safety profile in the context of its low concentration and topical use.

Molecular docking studies confirmed the binding of PCEO constituents to the AaegOBP1 binding site, with allohimachalol, β-elemene, aciphyllene and α-guaiene exhibiting binding affinities equal to or higher than DEET. In the AgamOBP1 binding site, α-guaiene showed a binding affinity comparable to DEET, suggesting a potential dual mode of action at both AaegOBP1 and AgamOBP1 receptors. These *in silico* predictions were consistent with the *in vivo* bioassays in adult *A. aegypti*, which demonstrated a marked repellent effect of the cream formulation containing PCEO at 200 ppm, providing 100% protection for up to 180 min.

Taken together, the phytochemical characterization, *in silico* predictions and *in vivo* repellency data support the potential of PCEO—particularly patchouli alcohol and selected sesquiterpenes—as a functional active ingredient in topical mosquito-repellent formulations. Although hepatotoxicity, carcinogenicity and skin-irritancy alerts were predicted for some constituents at systemic exposure levels, the negative Ames mutagenicity predictions, the relatively high TD_50_ values for several molecules and the low concentration of PCEO in the cream formulation support an acceptable preliminary safety profile for dermal use. This integrated evidence indicates that PCEO-based cream formulations may represent a sustainable and promising alternative for the complementary control of *A. aegypti* and arboviral diseases, while underscoring the need for further targeted toxicological and clinical studies to consolidate their long-term safety.

## Supplementary Material



## Data Availability

The data supporting the findings of this study are available within the article and its Supporting Information.

## Notes

Presents the GC–MS chromatogram of the essential oil, indicating the relative abundance of the identified constituents (Figure S1); individual mass spectra corresponding to peaks 1–16, confirming the identification of the major and minor compounds detected by GC–MS analysis (Figures S2–S17); ^13^C spectral data of *P. cablin* (Blanco) Benth essential oil (Figure S18); and complete ^13^C NMR spectral data of the crude essential oil, including experimental chemical shifts and comparative values from the literature (Table S3) (PDF)
