# Supplementary material for: Development and In Silico/In Vivo Evaluation of a Pogostemon cablin Essential Oil Cream as a Repellent against Aedes aegypti
Source: ACS Omega. 2026 Mar 30;11(13):21274–86. doi: 10.1021/acsomega.6c00802 (PMC13062987; doi:10.1021/acsomega.6c00802)
Supplement: Supplementary file 1 [file ao6c00802_si_001.pdf]

Development and *in silico*/*in vivo* Evaluation of a *Pogostemon cablin* Essential Oil Cream as a Repellent Against *Aedes aegypti*.

Lizandra Lima Santos[1], Lethicia Barreto Brandão[2], Alex Bruno Lobato Rodrigues[1], Rosany Lopes Martins[2], Anderson Luiz Pena da Costa[2], Cleidjane Gomes Faustino[2], Fernando Antônio de Medeiros[2], Sheylla Susan Moreira da Silva de Almeida[2]

[1]Department of Exact and Technological Sciences, Federal University of Amapá, 68902280, Macapá, Amapá, Brazil.

[2]Department of Biological and Health Sciences, Federal University of Amapá, 68902280, Macapá, Amapá, Brazil.

**Corresponding author*

Email: lizandralsantos@gmail.com

SUPPLEMENTARY FORMATION

Figure S1 – Chromatogram of the essential oil of *Pogostemon cablin (Blanco) Benth*

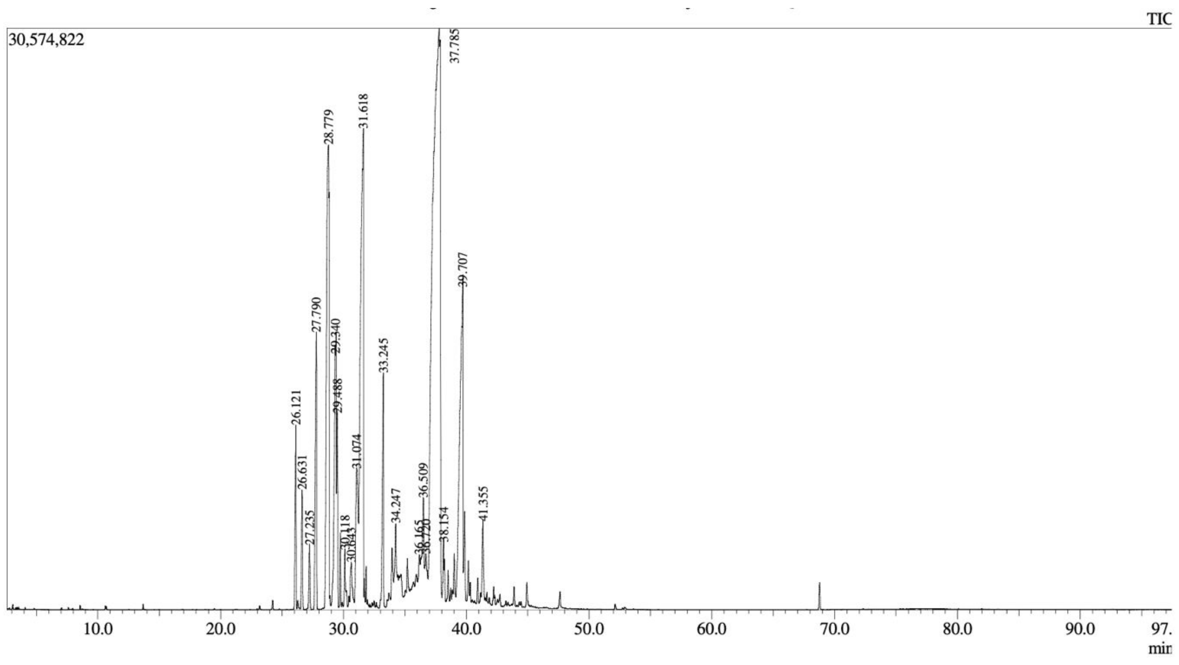

Figure S2 – Mass spectrum of peak 1 identified as substance β-patchoulene compared with literature

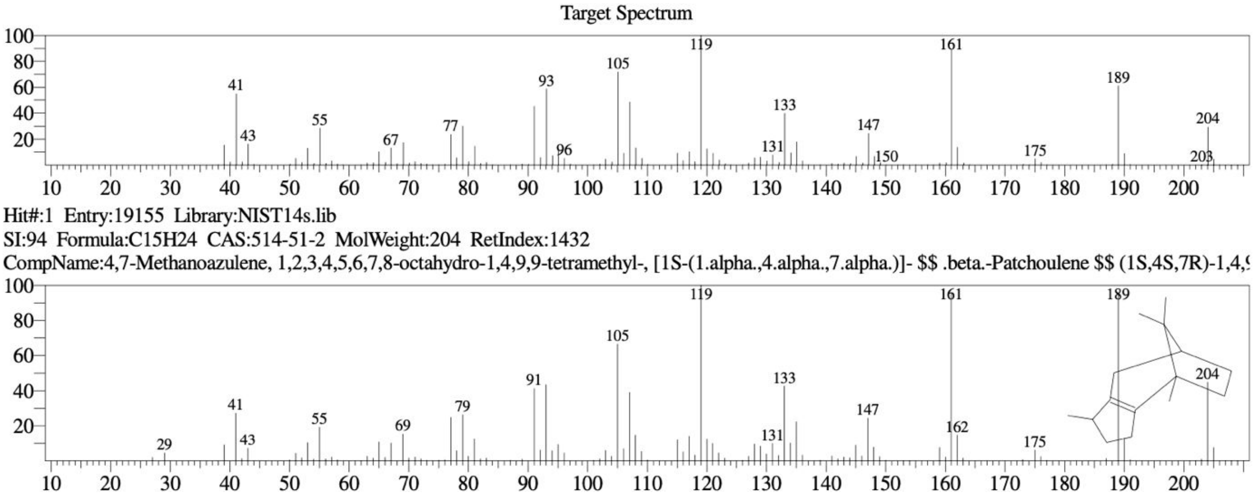

Source: Author; Substance β-patchoulene

Figure S3 – Mass spectrum of peak 2 identified as Substance β-elemene compared with literature

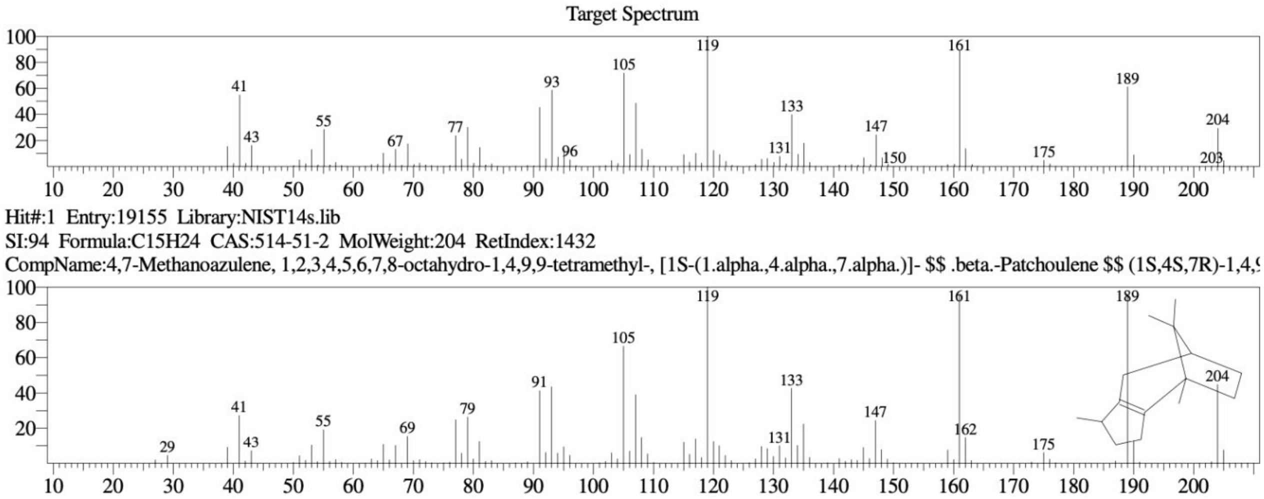

Source: Author; Substance β-elemene

Figure S4 – Mass spectrum of peak 3 identified as Substance Cycloseychellene compared with literature

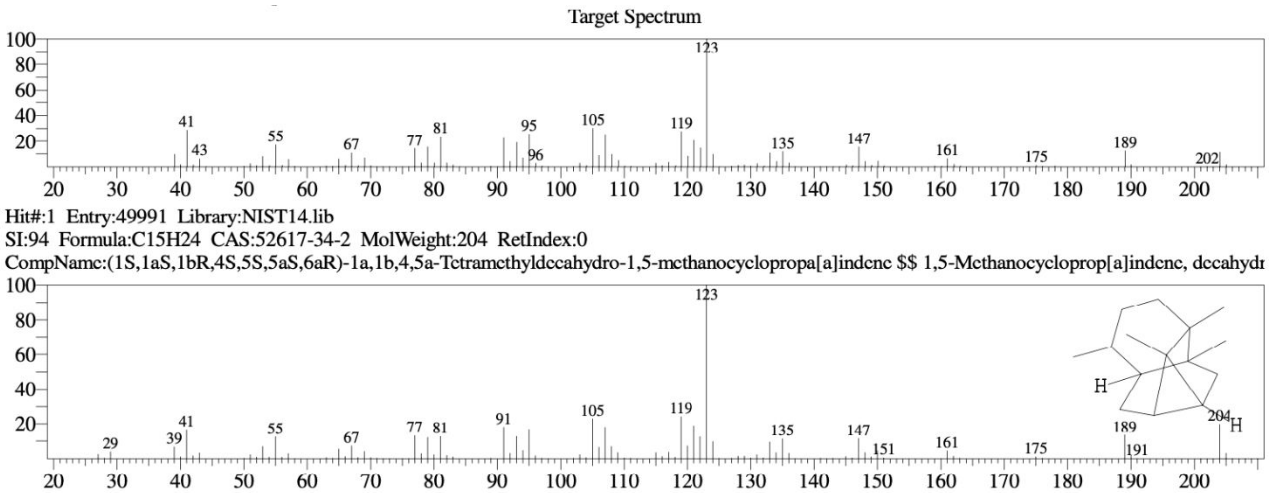

Source: Author; Substance Cycloseychellene

Figure S5 – Mass spectrum of peak 4 identified as Substance (*E*)- caryophyllene compared with literature

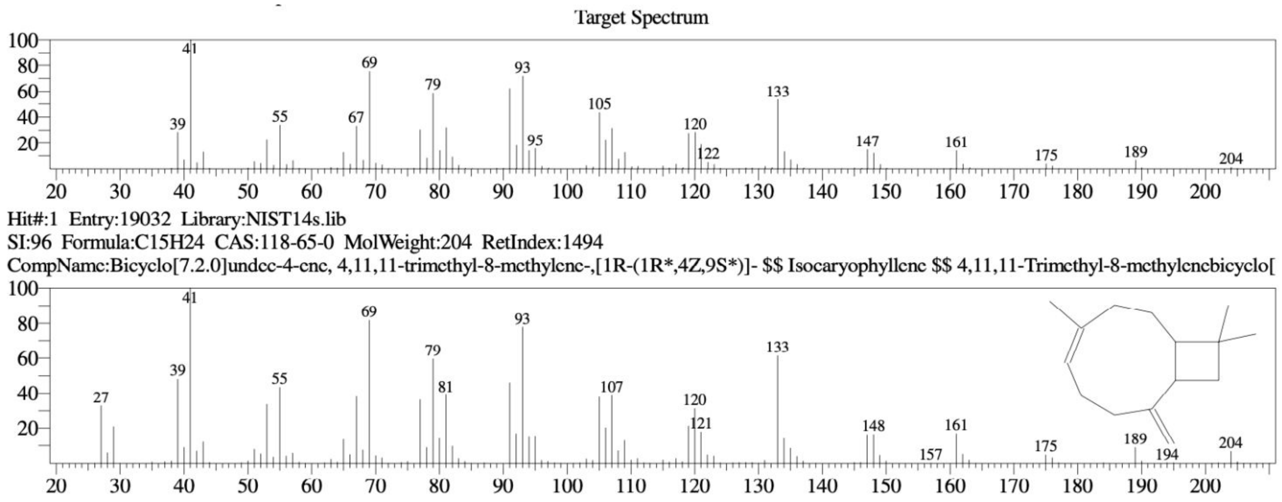

Source: Author; Substance (*E*)- caryophyllene

Figure S6 – Mass spectrum of peak 5 identified as Substance α-guaiene compared with literature

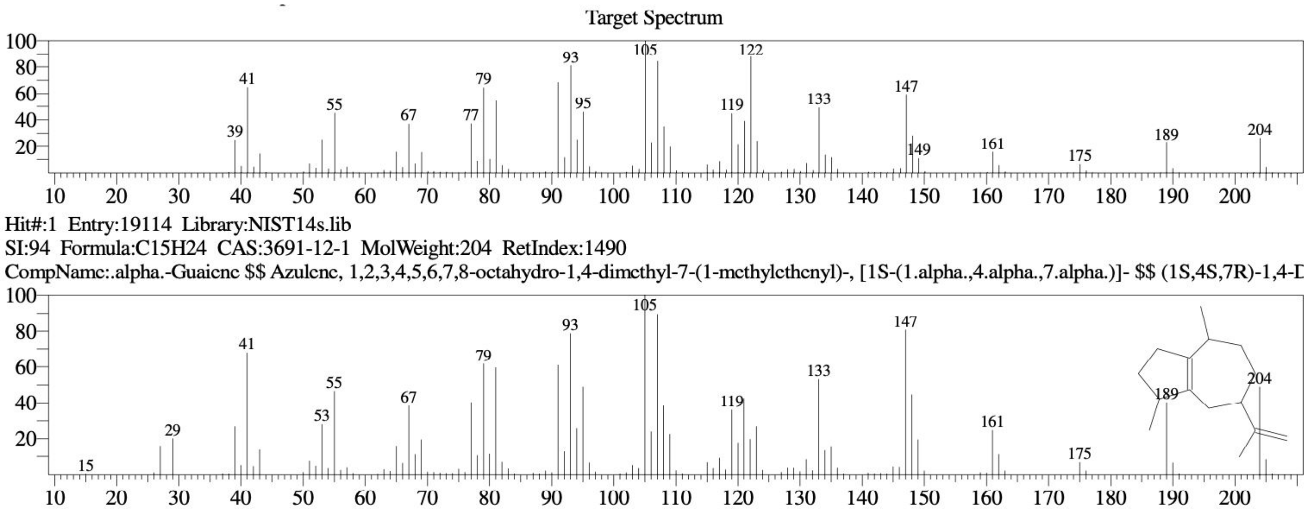

Source: Author; Substance α-guaiene

Figure S7 – Mass spectrum of peak 6 identified as Substance (E)-*Y*-bisabolene compared with literature

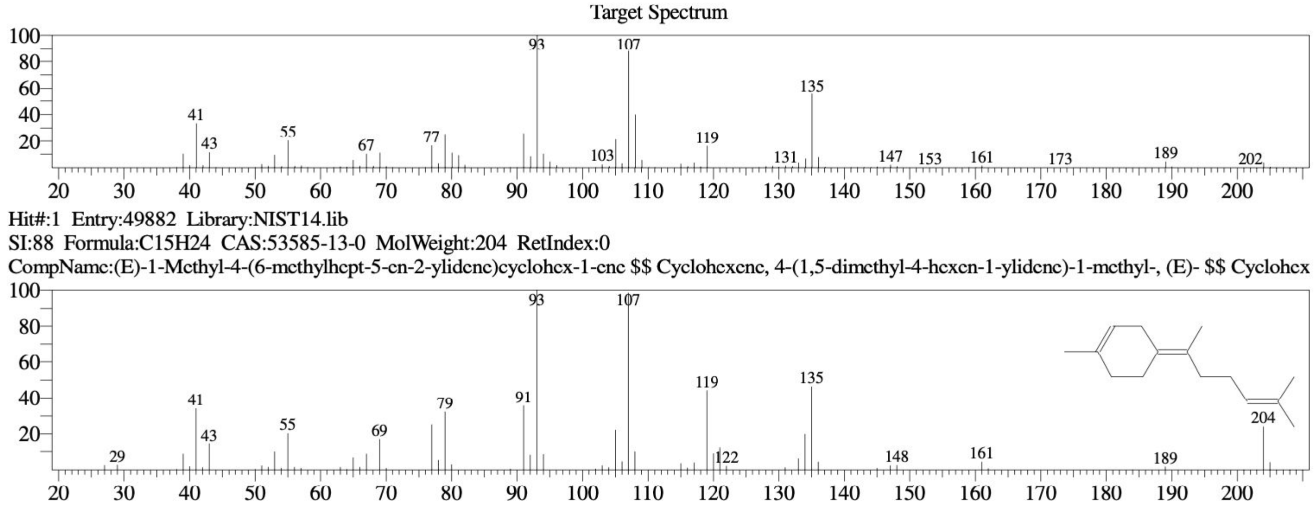

Source: Author; Substance (E)-*Y*-bisabolene

Figure S8– Mass spectrum of peak 7 identified as Substance α-bulnesene compared with literature

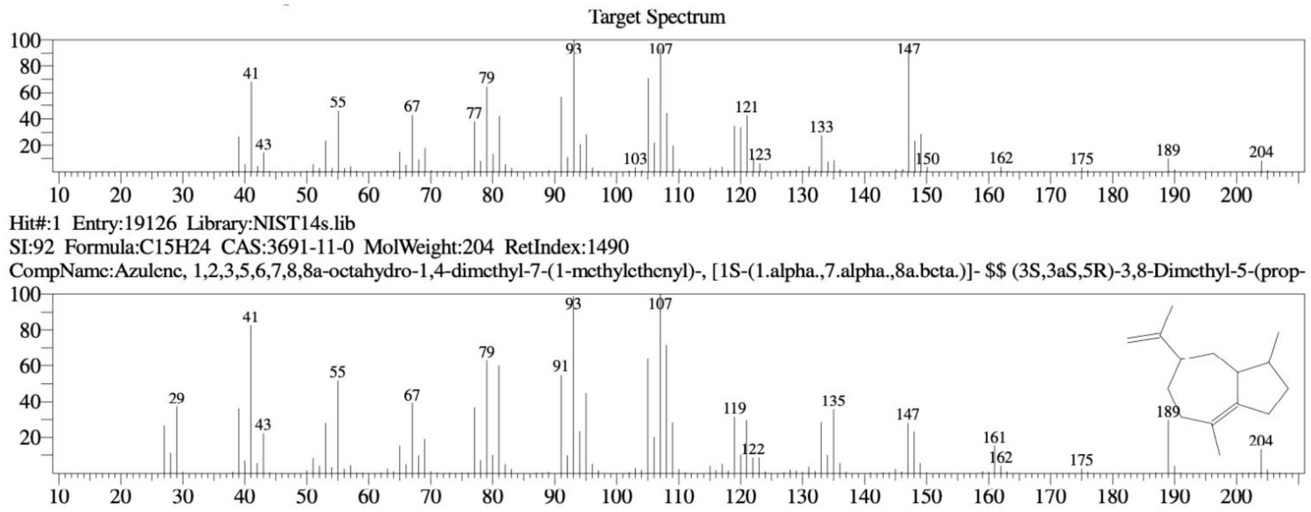

Source: Author; Substance α-bulnesene

Figure S9– Mass spectrum of peak 8 identified as Substance *y*- patchoulene compared with literature

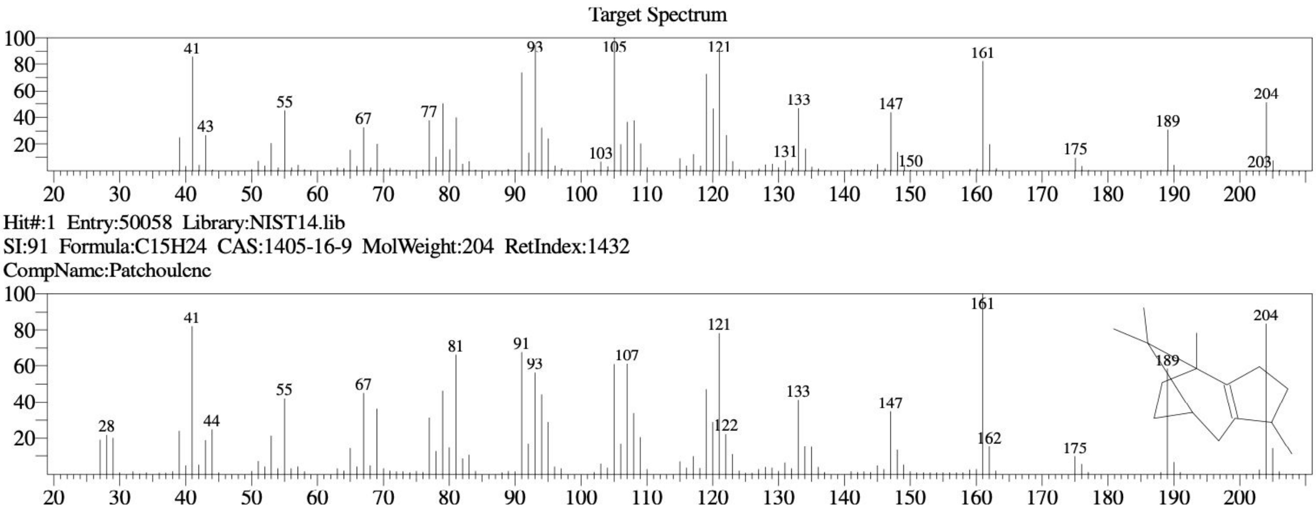

Source: Author; Substance *y*- patchoulene

Figure S10– Mass spectrum of peak 9 identified as Substance Aciphyllene compared with literature

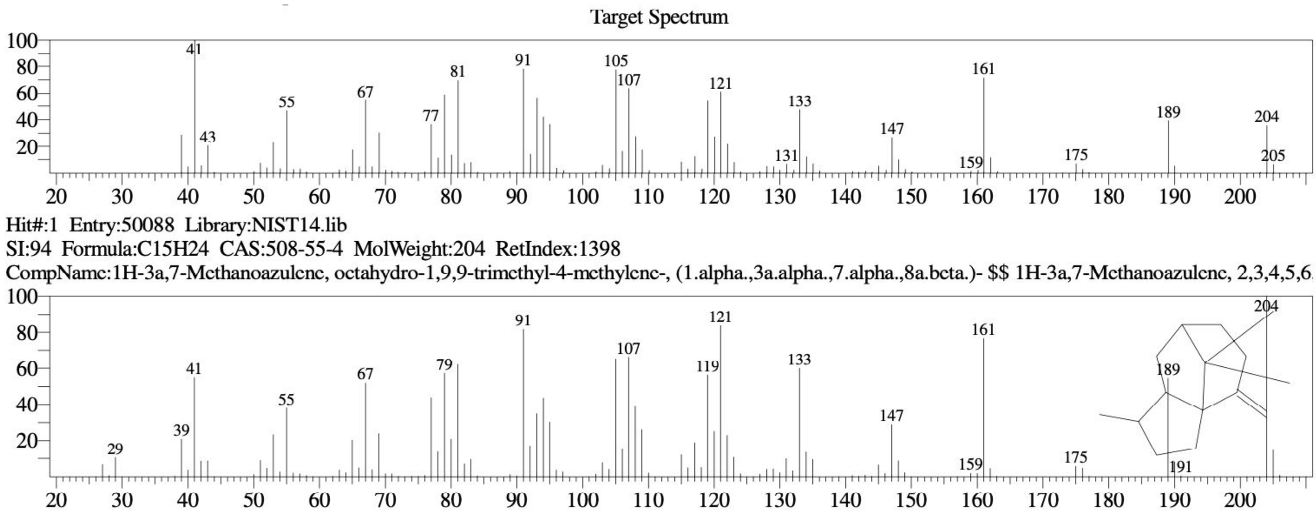

Source: Author; Substance Aciphyllene

Figure S11– Mass spectrum of peak 10 identified as Substance Norpatchoulenol compared with literature

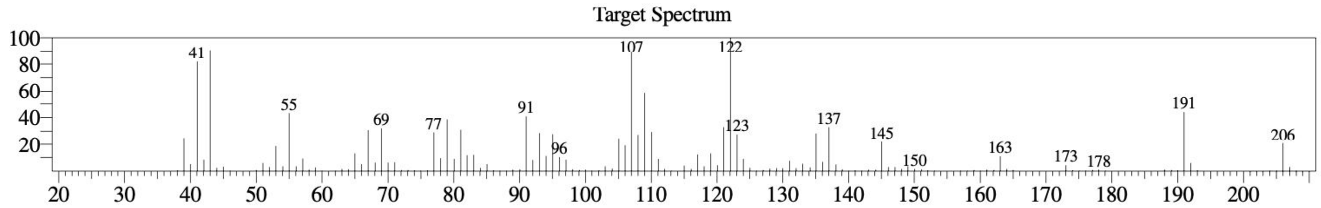

Hit#:1 Entry:51498 Library:NIST14.lib
SI:89 Formula:C14H22O CAS:41429-52-1 MolWeight:206 RetIndex:0
CompName:(1R,4aS,6R,8aS)-8a,9,9-Trimcthyl-1,2,4a,5,6,7,8,8a-octahydro-1,6-mcthanonaphthalcn-1-ol $$ Norpatchoulcnol $$ (+)-Norpatchoulcnol $$ 1,6

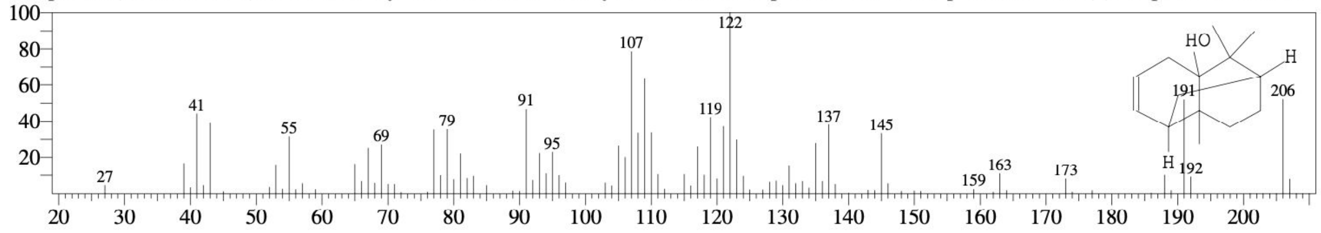

Source: Author; Substance Norpatchoulenol

Figure S12– Mass spectrum of peak 11 identified as Substance caryophyllene oxide compared with literature

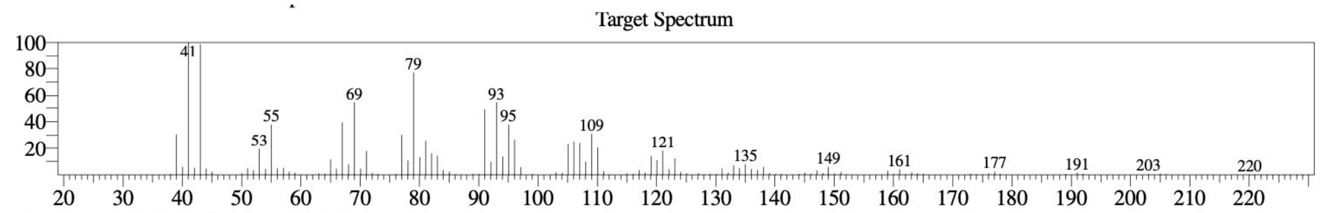

Hit#:1 Entry:62421 Library:NIST14.lib
SI:92 Formula:C15H24O CAS:1139-30-6 MolWeight:220 RetIndex:1507
CompName:Caryophyllcnc oxidc $$ 5-Oxatricyclo[8.2.0.0(4,6)-]dodccanc, 4,12,12-trimcthyl-9-mcthylcnc-, [1R-(1R*,4R*,6R*,10S*)]- $$ 5-Oxatricyclo(8.

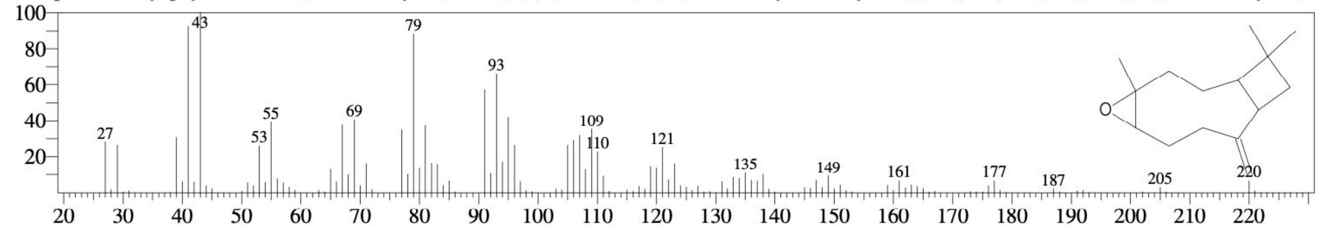

Source: Author; Substance caryophyllene oxide

Figure S13– Mass spectrum of peak 12 identified as Substance Palustrol compared with literature

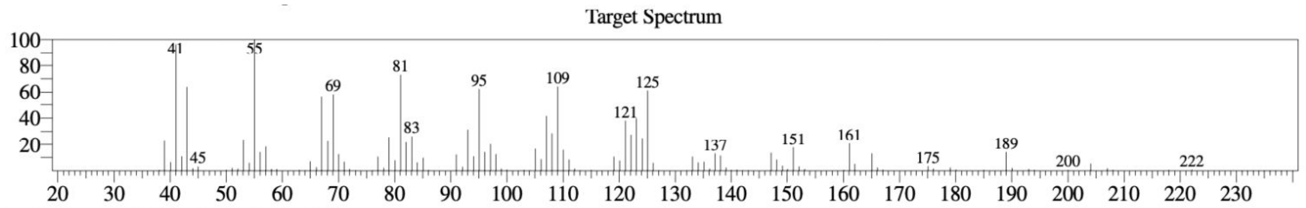

Hit#:1 Entry:64386 Library:NIST14.lib
SI:84 Formula:C15H26O CAS:95975-84-1 MolWeight:222 RetIndex:1530
CompName:4aH-cycloprop[c]azulcn-4a-ol, dccahydro-1,1,4,7-tctramcthyl- $$ 1,1,4,7-Tctramcthyldccahydro-4ah-cyclopropa[c]azulcn-4a-ol # $$

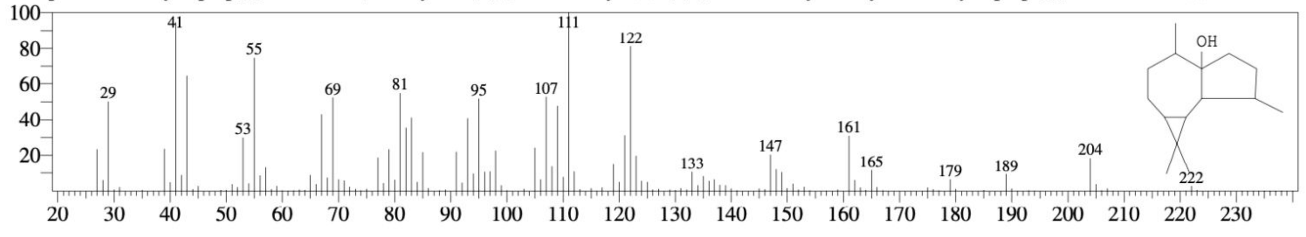

Source: Author; Substance Palustrol

Figure S14– Mass spectrum of peak 13 identified as Substance Spathulenol compared with literature

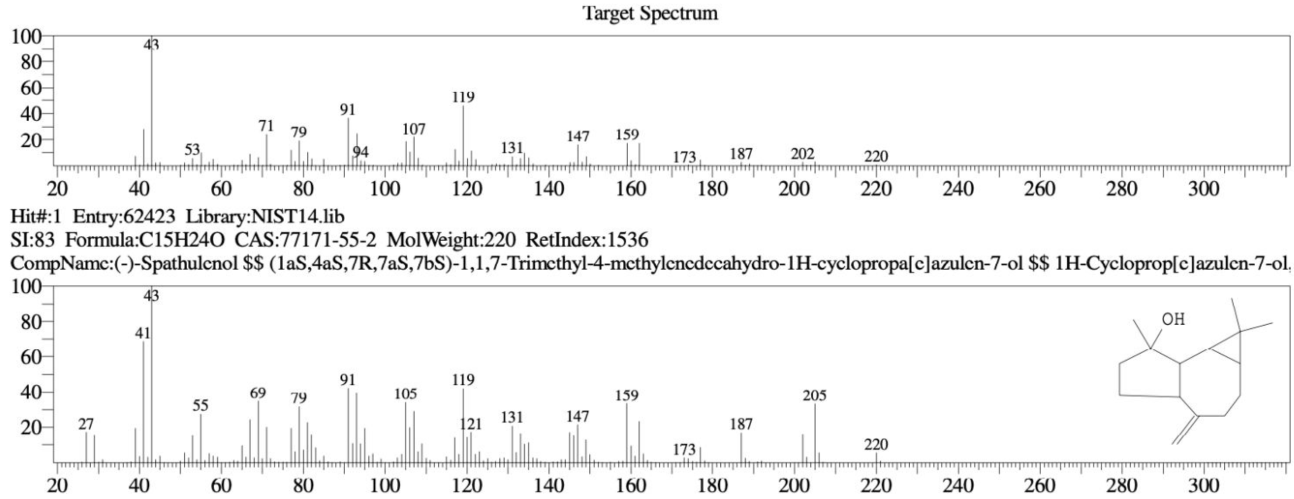

Source: Author; Substance Spathulenol

Figure S15– Mass spectrum of peak 14 identified as Substance Patchouli alcohol compared with literature

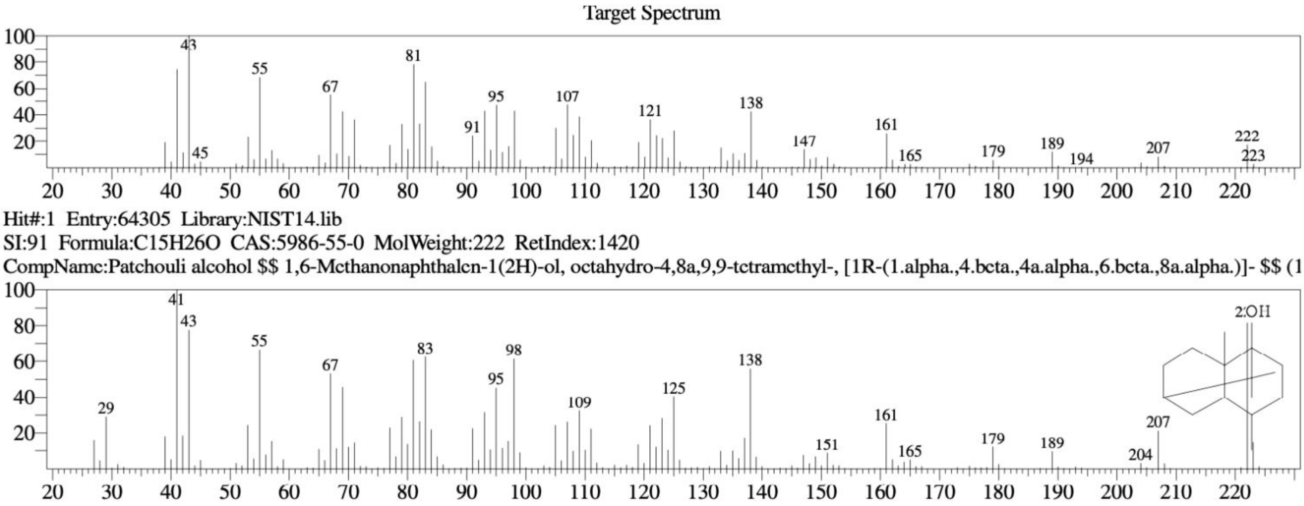

Source: Author; Substance Patchouli alcohol

Figure S16– Mass spectrum of peak 15 identified as Substance Allohimachalol compared with literature

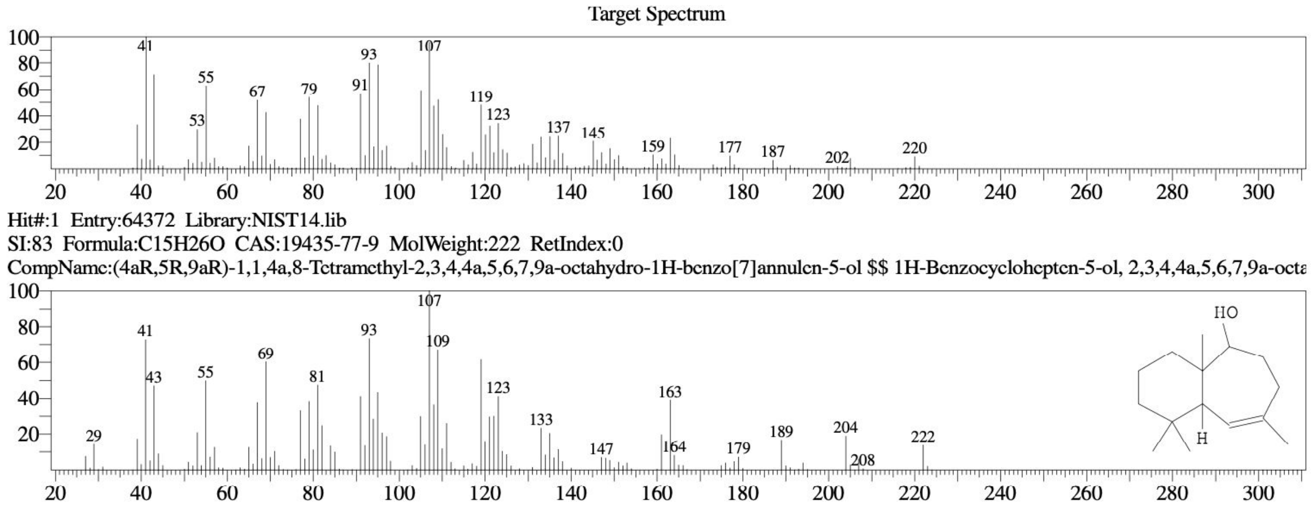

Source: Author; Substance Allohimachalol

Figure S17– Mass spectrum of peak 16 identified as Substance Pogostone compared with literature

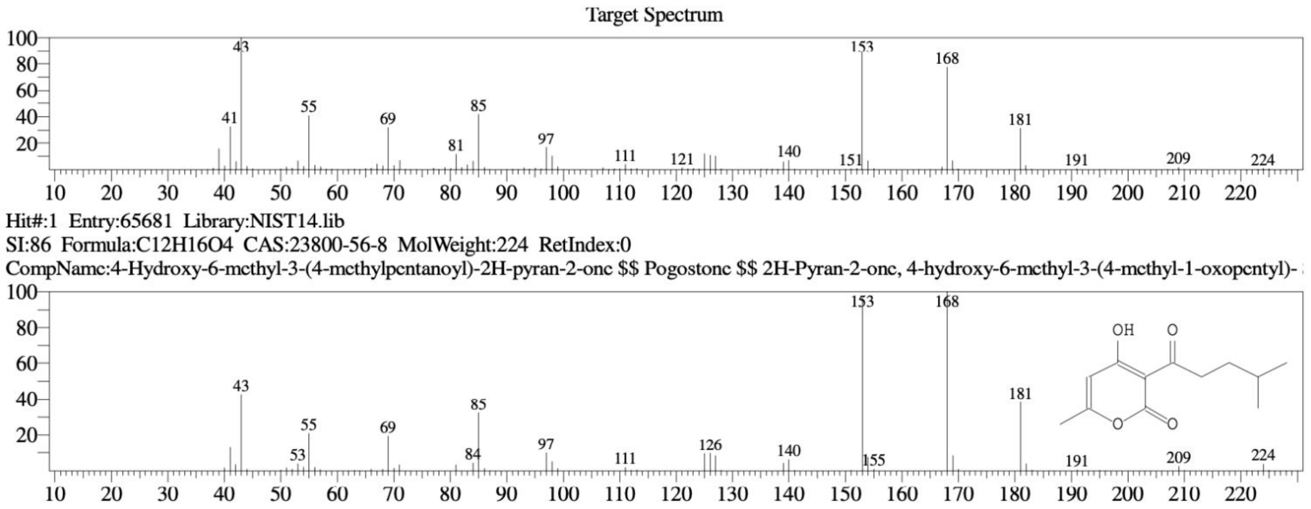

Source: Author; Substance Pogostone

S18 – ^{13}C Spectral Data of Pogostemon cablin (Blanco) Benth Essential Oil

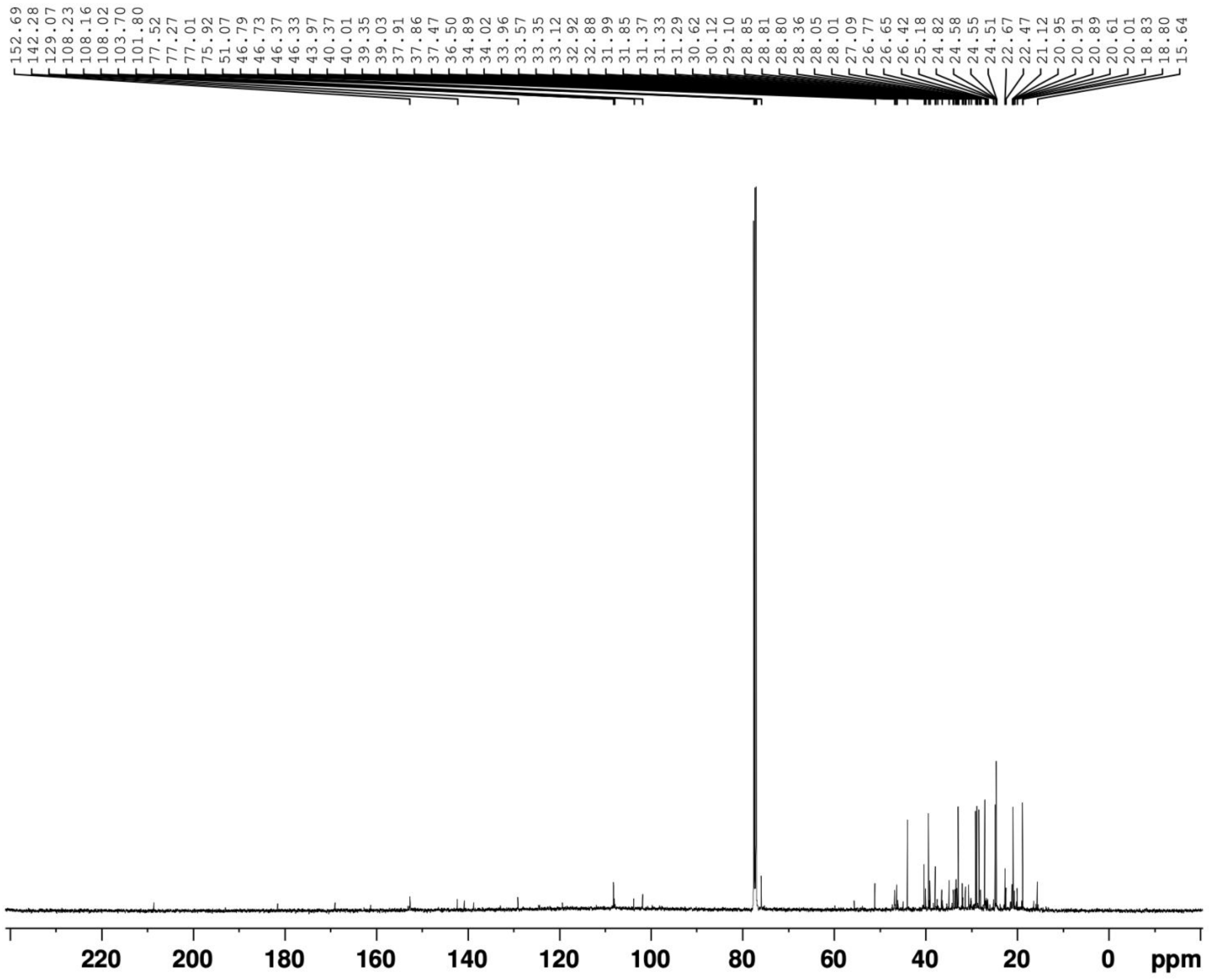

Current Data Parameters
NAME Nr.37687#1
EXPNO 2
PROCNO 1

F2 - Acquisition Parameters
Date_ 20210214
Time 23.26 h
INSTRUM spect
PROBHD Z816101_0032 (
PULPROG zgpg30
TD 32768
SOLVENT CDCl3
NS 12288
DS 4
SWH 32894.738 Hz
FIDRES 2.007735 Hz
AQ 0.4980736 sec
RG 203
DW 15.200 usec
DE 10.00 usec
TE 298.1 K
D1 2.00000000 sec
D11 0.03000000 sec
TD0 1
SFO1 125.7716219 MHz
NUC1 13C
P0 4.00 usec
P1 12.00 usec
PLW1 164.00000000 W
SFO2 500.1320005 MHz
NUC2 1H
CPDPRG[2 waltz16
PCPD2 80.00 usec
PLW2 9.80000019 W
PLW12 0.09800000 W
PLW13 0.04929300 W

F2 - Processing parameters
SI 32768
SF 125.7577574 MHz
WDW EM
SSB 0
LB 2.00 Hz
GB 0
PC 1.40

Table 1. ^{13}C NMR data of *Pogostemon cablin* (Blanco) Benth compared with literature

Position	α-guaiene		α-bulnesene		patchouli alcohol		pogostone	
	δC ppm	δC ppm[1]	δC ppm	δC ppm[1]	δC ppm	δC ppm[2]	δC ppm	δC ppm[3]
**C-1**	140.80	140.60	142.28	142.00	75.92	74.70	208.59	207.64
**C-2**	36.33	36.30	30.31	30.40	33.35	33.30	181.57	181.02
**C-3**	31.33	31.30	33.12	33.20	29.10	29.10	169.04	168.72
**C-4**	46.37	46.60	38.28	38.90	24.58	28.60	161.23	158.97
**C-5**	138.73	138.70	46.33	46.30	44.96	44.96	101.80	100.23
**C-6**	33.57	33.40	32.92	32.90	25.18	25.18	101.80	99.41
**C-7**	46.73	46.70	51.07	50.90	39.41	39.70	38.28	38.64
**C-8**	31.29	31.20	31.99	32.00	25.94	25.20	31.99	31.98
**C-9**	33.57	34.00	33.89	33.80	29.20	29.50	27.09	27.02
**C-10**	33.89	33.90	129.07	128.80	28.61	28.20	21.44	21.56
**C-11**	152.62	152.10	152.62	152.20	40.71	40.90	21.37	21.54
**C-12**	108.02	108.00	108.02	108.10	19.00	19.00	20.61	20.60
**C-13**	21.44	20.40	20.95	20.90	21.44	21.60		
**C-14**	18.80	18.60	22.47	22.20	24.82	24.80		
**C-15**	19.00	19.80	15.41	15.40	28.05	28.10		

[1] doi.org/10.1080/10412905.1997.10554251; [2] doi.org/10.1007/s11418-011-0550-x
[3] doi.org/10.1016/j.fitote.2015.08.003
